# Insights into Cisplatin Binding to Uracil and Thiouracils from IRMPD Spectroscopy and Tandem Mass Spectrometry

**DOI:** 10.1021/jasms.0c00006

**Published:** 2020-02-18

**Authors:** Davide Corinti, Maria Elisa Crestoni, Barbara Chiavarino, Simonetta Fornarini, Debora Scuderi, Jean-Yves Salpin

**Affiliations:** † Dipartimento di Chimica e Tecnologie del Farmaco, Università di Roma “La Sapienza”, P. le A. Moro 5, Roma 00185, Italy; ‡ Universite′ Paris-Saclay, CNRS, Institut de Chimie Physique UMR8000, Orsay 91405, France; § Université Paris-Saclay, CNRS, Univ Evry, LAMBE, Evry-Courcouronnes 91025, France; ∥ CY Cergy Paris Université, LAMBE, Evry-Courcouronnes 91025, France

**Keywords:** cisplatin, uracil, thiouracils, IRMPD spectroscopy, FT-ICR mass spectrometry

## Abstract

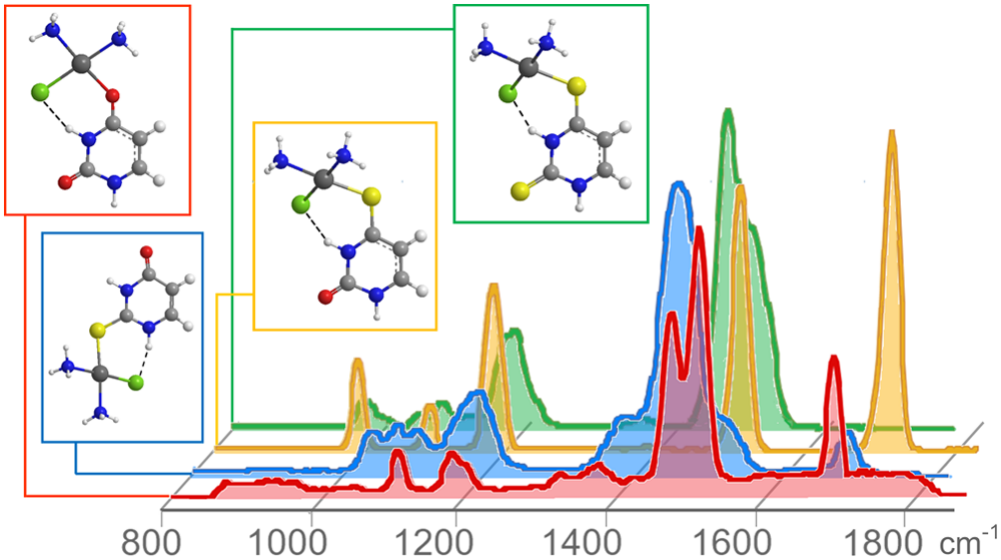

The monofunctional primary complexes *cis*-[PtCl(NH_3_)_2_(L)]^+^, formed by the reaction of cisplatin, a major chemotherapeutic agent, with four nucleobases L, i.e., uracil (U), 2-thiouracil (2SU), 4-thiouracil (4SU), and 2,4-dithiouracil (24dSU), have been studied by a combination of infrared multiple photon dissociation (IRMPD) action spectroscopy in both the fingerprint (900–1900 cm^–1^) and the N–H/O–H stretching (3000–3800 cm^–1^) ranges, energy-resolved collision-induced dissociation (CID) mass spectrometry, and density functional calculations at the B3LYP/LACVP/6-311G** level. On the basis of the comparison across the experimental features and the linear IR spectra of conceivable structures, the cisplatin residue is found to promote a monodentate interaction preferentially with the O4(S4) atoms of the canonical forms of U, 4SU, and 24dSU and to the S2 atom of 2SU, yielding the most stable structures. Additional absorptions reveal the presence of minor, alternative tautomers in the sampled ion populations of 2SU and 24dSU, underlying the ability of cisplatin to increase the prospect of (therapeutically beneficial) nucleic acid strand disorder. Implication of these evidence may provide insights into drug mechanism and design.

## Introduction

In spite of their peculiar biological properties, only a few theoretical and experimental studies dedicated to the structural characterization of thiouracils at the molecular level were available at the end of the 1980s. However, these molecules have since attracted significant attention. Thiouracils have been identified as minor components of transfer-RNA,^1^ and the existence of these molecules in many tautomeric forms, like for other nucleobases, seems to be a key point to account for the mistranslation of genetic information.^2−4^ Therefore, this feature has motivated both theoretical and experimental studies on tautomeric equilibria of nucleobases and thiobases in order to establish a relationship between the presence of enol tautomeric forms and the appearance of point mutations during DNA replication.^5−16^ In addition, thiouracils exhibit many interesting therapeutic applications for antithyroid,^17,18^ antiviral,^19,20^ anticancer,^21,22^ and heart disease^23,24^ therapies.

Due to the presence of soft thiocarbonyl group(s), thiouracils have also been selected to reveal and capture transition metal ions and notably mercury.^25,26^ However, despite strong and selective binding properties, gas-phase studies describing their interactions with metal ions at the molecular level are rather scarce. The effect of Cu^2+^ ions onto deprotonated forms of thiouracils has been inspected only theoretically,^27,28^ whereas the scrutiny of Pb^2+^/thiouracils^29^ and Ca^2+^/thiouracils^30,31^ complexes combined both experiments and calculations. More recently, infrared multiple photon dissociation (IRMPD) spectroscopy has been applied to the characterization of protonated and sodiated uracil and thiouracils complexes,^15,32,33^ disclosing that protonation preferentially stabilizes alternative, noncanonical tautomers of uracil and of all the thiouracils aside from 4-thiouracil (4SU), whereas the sodium cation binds to the canonical tautomers of uracil (U) and 2-thiouracil (2SU), and to minor tautomers of 4-thiouracil (4SU) and 2,4-thiouracil (24SU). Other contributions on the binding of Cu^+^/uracil and Ag^+^/uracil,^34^ aimed at determining if tautomeric forms and cation−π binding that may cause mismatch are involved in the complexation process, have been reported. Proceeding with growing complexity of the ligand, IRMPD spectra of protonated thiated uridines have been recently scrutinized unveiling the occurrence of protonation on the 4 position of hydroxyl–sulfhydryl or sulfhydryl–hydroxyl tautomeric structures.^35^

In the context of the ongoing interest in spectroscopic surveys on naked primary aqua intermediates^36^ of cisplatin (*cis*-diamminedichloroplatinum(II), a leading antitumor agent which has revolutionized cancer treatments, and on its complexes with prime targets including amino acids,^37−39^ purines,^40^ and nucleotides,^41,42^ a thorough description of cisplatin (cisPt) interaction with uracil (U), and its thio derivatives, namely 2-thiouracil (2SU), 4-thiouracil (4SU), and 2,4-dithiouracil (24dSU), is presented here based on mass spectrometry, IRMPD spectroscopy, and density functional calculations.

Our previous work on cisplatin binding to adenine and guanine^40^ has shown that Pt is attached to the N7 position of guanine and to the N3 and N1 atoms of adenine. Analogous coordination modes besides a macrochelate arrangement have emerged in the spectroscopic scrutiny of the cisPt adducts with the nucleic acid building blocks, 2′-deoxyguanosine-5′monophosphate^41^ and 2′-deoxyadenosine-5′monophosphate.^42^

Here, the presence of N, O, and S donor atoms in thiouracils may enlarge the variety of cisPt coordination targets. The present study aims to obtain a direct experimental characterization of *cis*-[PtCl(NH_3_)_2_(L)]^+^ ions, accessed by electrospray ionization (ESI), so as to identify the preferred metal binding site, to define the various (non)canonical tautomeric forms of uracil and three thiouracils (L = 2SU, 4SU, 24dSU) and to elucidate the effect of thio-keto substitution onto the complexation process.

Unveiling the intrinsic properties of metal-containing therapeutics may contribute to elucidate their mechanisms of actions and to spur new directions in the design of anticancer drugs.^43^

## Experimental Section

### Materials

Cisplatin, uracil, and thiouracils were purchased from Sigma-Aldrich (Sigma-Aldrich s.r.l. Milan, Italy) and used without further purification. To generate the complexes of interest, an aqueous solution of cisplatin ca. 1 × 10^–3^ M, allowed to stand overnight, was mixed with a solution of the selected (thio)uracil nucleobases (L= U, 2SU, 4SU, 24dSU) and diluted with water. Final solutions of the two analytes in isomolar ratio and concentration of 5 × 10^–5^ M in methanol/water (1:1 v/v) were infused into the ESI source.

### CID Experiments

Energy-variable collision induced dissociation (CID) experiments have been recorded on a commercial hybrid triple-quadrupole linear ion trap mass spectrometer (2000 Q-TRAP Applied Biosystems) with a Q1q2Q_LIT_ configuration (Q1, first mass analyzing quadrupole; q2, nitrogen-filled collision cell; Q_LIT_, linear ion trap) equipped with an ESI source. After desolvation, electrosprayed [*cis*-[PtCl(NH_3_)_2_(L)]^+^ ions were mass-selected (Q1) and allowed to collide with N_2_ (nominal pressure of 1.1 × 10^–5^ mbar) in q2 at variable collision energy (*E*_LAB_ = 5–50 eV). The product ions were monitored by scanning Q_LIT_. Typical experimental parameters were: ion spray voltage at 5500 V, curtain gas set at 20 psi, GS1 at 20 psi, declustering potential at 40 V, and entrance potential at 5 V. Quantitative absolute threshold energies are by no means directly amenable. However, phenomenological dissociation threshold energies (TEs) and comparative assessment for different fragmentation channels can be attained from the linear interpolation of the rise of breakdown curves by converting the collision energies to the center of mass frame *E*_CM_ = [*m*/(*m* + *M*)]*E*_LAB_, where *m* and *M* are the masses of the collision gas and of the ion, respectively. Corrections for the nominal zero energy were acquired from retarding potential (RP) experiments, in which the intensity of the parent ion is plotted as a function of the entrance potential.^44^

### IRMPD Experiments

IRMPD experiments were performed in two distinct energy ranges. The vibrational modes associated with the XH (X = C, N, O) stretches in the 3100–3800 cm^–1^ frequency range were recorded by means of an optical parametric oscillator/amplifier (OPO/OPA, LaserVision) laser system coupled to a Paul ion trap mass spectrometer (Esquire 6000+, Bruker Daltonics), as already described.^45,46^ The typical output energy from the OPO/OPA laser operated at 10 Hz was 20–24 mJ/pulse, with a spectral bandwidth of about 3–4 cm^–1^. In the trap, ions were accumulated for 10 ms and then mass-selected prior to IR irradiation for 500 ms. The mass spectrum was typically derived from an accumulation of four scans. To improve the IRMPD signal intensity, the sampled ions were irradiated by using an auxiliary, CO_2_ laser (Universal Laser Systems, Inc., Scottsdale, AZ). A 20 ms long CO_2_ pulse of 13 W, corresponding to an energy of 260 mJ, followed each OPO/OPA pulse, delayed by 10 μs.^47^

The fingerprint region (800–2000 cm^–1^) was explored using the beamline of the free electron laser (FEL) of the Centre Laser Infrarouge d’Orsay (CLIO).^48,49^The electron energy of the FEL was set at 44.4 MeV to optimize the laser power in the frequency region of interest and ensure a fairly stable laser power (900–1100 mW). IR light was delivered in 9 μs macropulses (25 Hz), each containing 600 micropulses (0.5–3 ps long). Typical macropulse energies are ca. 40 mJ. For the present study, the FEL beamline was admitted into the cell of a hybrid FT-ICR tandem mass spectrometer (APEX-Qe Bruker) equipped with a 7.0 T actively shielded magnet and coupled to a quadrupole-hexapole interface for mass-filtering and ion accumulation. The complexes of interest were first mass-selected in the quadrupole and then accumulated and collisionally cooled for 300 ms in the presence of a buffer gas (argon) in the linear hexapole, prior to their transfer into the ICR cell. Isolated charged complexes were then irradiated for 250–500 ms with the IR FEL light, after which the resulting ions are mass-analyzed.^49,50^ To avoid saturation effects of the most intense absorptions, IRMPD spectra were also recorded using one attenuator to decrease the irradiation power by a factor of 3.

The IRMPD spectra presently reported correspond to the photofragmentation yield *R* (*R* = −ln[*I*_precursor_/(*I*_precursor_ + ∑*I*_fragment_)], where *I*_precursor_ and *I*_fragment_ are the integrated intensities of the mass peaks of the precursor and of the fragment ions, respectively) as a function of the photon wavenumber.^49,51^

### Computational Studies

Density functional calculations were carried out using the hybrid B3LYP density functional,^52,53^ as implemented in the Gaussian-09 set of programs.^54^ Geometry optimization was achieved without any symmetry constraint using the 6-311G** basis set. In order to describe the Pt atom, we combined the Los Alamos effective core potential (ECP) with the LACV3P** basis set.^55−57^ Harmonic vibrational frequencies were also computed at this level to estimate the zero-point vibrational energy (ZPE) corrections and to characterize the stationary points as local minima or saddle points.

The infrared absorption spectra of the various structures were calculated within the harmonic approximation. Calculated frequencies were scaled by a factor of 0.974 in the fingerprint region and by 0.957 in the X-H stretch region, for a better agreement with the experimental spectrum, as detailed in a previous study about the *cis*-[PtCl(NH_3_)_2_G]^+^ complex.^40^

## Results and Discussion

### Photodissociation and CID Experiments

A great deal of effort has been been devoted so far in examining the stability and binding interactions of uracil and its thio and halo derivatives by means of energy-resolved CID experiments^29,58−61^ and chemical dynamics simulations^62,63^ and statistical reactivity approaches.^64^

**Figure 1:**
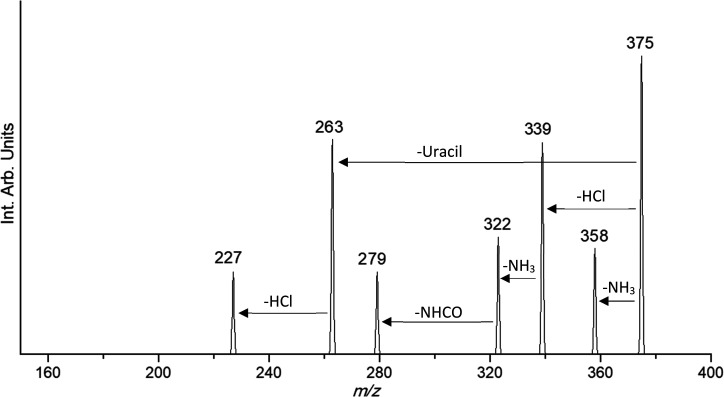
Collision-induced dissociation mass spectrum of *cis*-[PtCl(NH_3_)_2_(U)]^+^ at *m*/*z* 375 obtained at collision (lab) energy (*E*_LAB_) of 5 eV in a hybrid triple-quadrupole linear ion trap mass spectrometer (QTrap API 2000). At this *E*_LAB_ value a considerable fraction of the precursor ion has undergone fragmentation by loss of NH_3_, a process occurring already in the region preceding the collision quadrupole.

Here, the complexes of cisplatin with uracil and thiouracil nucleobases, *cis*-[Pt(CH_3_)_2_Cl(L)]^+^ (L = U, 2SU, 4SU, 24dSU) were mass-isolated and submitted to CID to obtain information about structural and thermodynamic features. The observed fragments are reported in Table 1 together with the values of phenomenological dissociation threshold (TE) obtained from breakdown curves. Overall, the four complexes present a similar dissociation pattern involving mainly the ammonia ligands and the remaining chlorido ligand eliminated as hydrogen chloride. The complexes presenting either U, 2SU, or 4SU as ligands give two additional fragmentation pathways, including the loss of NHCO, which originates from the fragmentation of the nucleobase ring, and the elimination of the corresponding neutral nucleobase (*m*/*z* 263), likely by direct cleavage of the L–Pt bond. However, the latter fragmentation is much more prevalent for *cis*-[PtCl(NH_3_)_2_(U)]^+^, in which case this channel represents one of the most abundant dissociation path even at low collision energy (Figure 1). Indeed, the lower percentage of intact 2SU and 4SU cleavage from the corresponding cisPt complexes, as well as the absence of the corresponding dissociation path from *cis-*[PtCl(NH_3_)_2_(24dSU)]^+^ (Figures S1–S3) are in agreement with the well-known bias for Pt binding to S-containing nucleophiles, which increases the energetic demand for breaking the Pt–L bond.^43^

**Table 1:** Observed IRMPD and CID Dissociation Channels from *cis*-[PtCl(NH_3_)_2_(L)]^+^ Complexes and Phenomenological Thresholds (kJ mol^–1^)

*cis*-[PtCl(NH_3_)_2_(L)]^+^	reactant ion (*m*/*z*)	product ions^a^ (*m*/*z*)	neutral loss	TE^b^
L = U	375	358	NH_3_	na^c^
		339	HCl	
		322	NH_3_, HCl	
		**279**	NH_3_, HCl, NHCO	
		**263**	U	
		**246**	U and NH_3_	
		**227**	U, HCl	
L = 2SU	391	374	NH_3_	89
		338	NH_3_, HCl	
		**321**	2 NH_3_, HCl	
		**295**	NH_3_, HCl, NHCO	
		**263**	2SU	
L = 4SU	391	374	NH_3_	89
		338	NH_3_, HCl	
		**321**	2 NH_3_, HCl	
		**295**	NH_3_, HCl, NHCO	
		**263**	4SU	
L = 24dSU	407	390	NH_3_	75
		354	NH_3_, HCl	
		**337**	2 NH_3_, HCl	

a Fragments observed only in CID mass spectra are in boldface.

b Phenomenological threshold energies (TE) in kJ mol^–1^ are given with ±5–10 kJ mol^–1^ uncertainty.

c na stands for not available.

The breakdown curves together with the RP experiments are reported in Figures S4–S6 in the SI, with the exception of the *cis*-[PtCl(NH_3_)_2_(U)]^+^ complex, whose low absolute intensity hampered obtaining meaningful data. The phenomenological TE values for the remaining complexes were found to be equal to 89 ± 5–10, 89 ± 5–10, and 75 ± 5–10 kJ mol^–1^ for 2SU, 4SU, and 24dSU, respectively. These values, obtained by summing the abundances of all fragments, allow a comparative evaluation for the lowest energy dissociation route, namely the cleavage of ammonia in the *trans* position with either the chlorido or the nucleobase ligand. In four-coordinate planar complexes, the threshold energy for breaking a metal–ligand bonds is influenced by the nature of the ligand *trans* to the leaving group. As a result, the same TE values obtained for 2SU and 4SU complexes, which therefore are barely differentiated by CID measurements, suggest these nucleobases bind platinum rather strongly and at a site with akin electronic features. The slightly smaller TE value (by 14 kJ mol^–1^) observed for *cis*-[PtCl(NH_3_)_2_(24dSU)]^+^ is consistent with 24dSU interacting in a similar way with platinum and suggests a second thio-keto substitution to somewhat enhance the *trans* influence of the ligand on the ammonia loss.

The same lowest energy fragmentation routes by loss of NH_3_ and HCl occur when *cis*-[PtCl(NH_3_)_2_(L)]^+^ ions are assayed by IRMPD spectroscopy in both the explored fingerprint and NH/OH stretching ranges, while the higher energy-demanding L elimination is not detected.

This evidence is in agreement with the notion that both multiple photon absorption and low–energy CID are slow heating processes that promote fragmentation along the lowest energy channel.^65,66^

Exemplary mass spectra obtained when mass-selected *cis*-[PtCl(NH_3_)_2_(L)]^+^ are irradiated with the CLIO FEL light tuned at 1810 (L = U), 1490 (2SU), 1280 (L = 4SU) and 1285 (L = 24dSU) cm^–1^ are illustrated in Figures S7–S10, respectively.

### IRMPD Spectra

**Figure 2:**
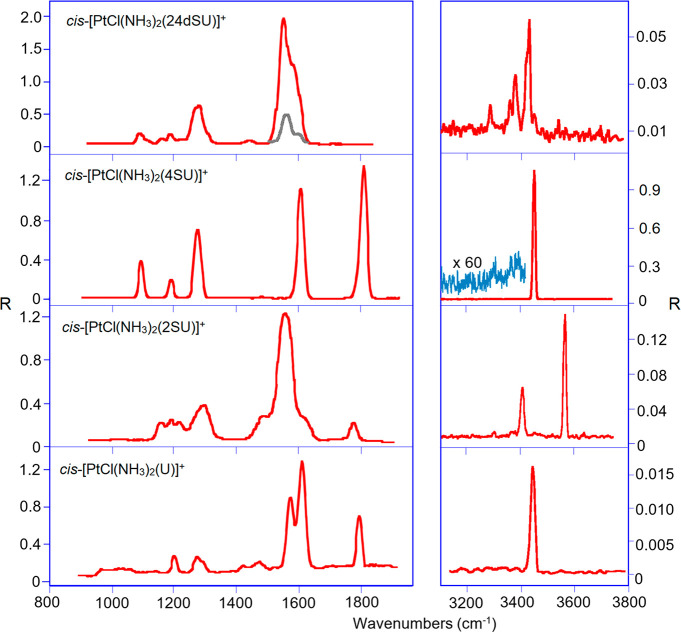
Experimental IRMPD spectra of *cis*-[PtCl(NH_3_)_2_L]^+^ (from bottom to top L = U, 2SU, 4SU, 24dSU) recorded in the fingerprint and in the NH/OH stretch ranges.

The potential of IRMPD spectroscopy to elucidate structural features and to distinguish isomers/conformers of a wide variety of (bio)molecular ions has been taken advantage of in the present study.^47,67−69^ In the mid-IR range, the IRMPD spectra of *cis*-[PtCl(NH_3_)_2_(L)]^+^ (L = U, 2SU, 4SU, 24dSU) presented in Figure 2 exhibit distinct profiles, where prominent absorptions at high wavenumber values highlight differences ascribable to the thio–keto substitution. While two strong, poorly resolved bands at 1580 and 1615 cm^–1^, a sharp peak at 1800 cm^–1^ and two small absorptions at 1426 and 1480 cm^–1^ are recorded for *cis*-[PtCl(NH_3_)_2_(U)]^+^, an intense broad peak centered at 1560 cm^–1^ dominates the spectrum of *cis*-[PtCl(NH_3_)_2_(2SU)]^+^ with two shoulders on both red and blue sides at 1488 and 1619 cm^–1^ and a very small absorption, likely due to a carbonyl stretch, at 1770 cm^–1^. With regard to *cis*-[PtCl(NH_3_)_2_(4SU)]^+^, two sharp strong peaks appear at 1609 and 1808 cm^–1^, whereas the IRMPD spectrum of *cis*-[PtCl(NH_3_)_2_(24dSU)]^+^ exhibits an intense broad feature, whose partial resolution in bands centered at 1554 and 1586 cm^–1^ has been attained at an attenuated laser pulse energy of ca. 14 mJ/pulse. Below 1300 cm^–1^, *cis*-[PtCl(NH_3_)_2_(4SU)]^+^ presents three sharp peaks at 1098, 1193, and 1279 cm^–1^ which find a counterpart in (i) two small absorptions at 1209 and 1290 cm^–1^ for *cis*-[PtCl(NH_3_)_2_(U)]^+^; (ii) an envelope of bands between 1145 and 1240 cm^–1^ and an intense and broad one at 1297 cm^–1^ for *cis*-[PtCl(NH_3_)_2_(2SU)]^+^; three weak bands at 1094, 1123–1163 and 1194 cm^–1^ and an intense feature at 1280 cm^–1^ for *cis*-[PtCl(NH_3_)_2_(24dSU)]^+^.

In the NH/OH stretching range, all IRMPD spectra are dominated by a strong band at 3450 (L = U), 3569 (L = 2SU), 3452 (L = 4SU), and 3431 (L= 24dSU) cm^–1^, along with weaker bands at 3405 cm^–1^ for *cis*-[PtCl(NH_3_)_2_(2SU)]^+^ and at 3285, 3380, and 3452 cm^–1^ for *cis*-[PtCl(NH_3_)_2_(24dSU)]^+^.

### Structural Characterization of the *cis*-[PtCl(NH_3_)_2_(L)]^+^ Complexes

#### Computational Study

**Figure 3:**
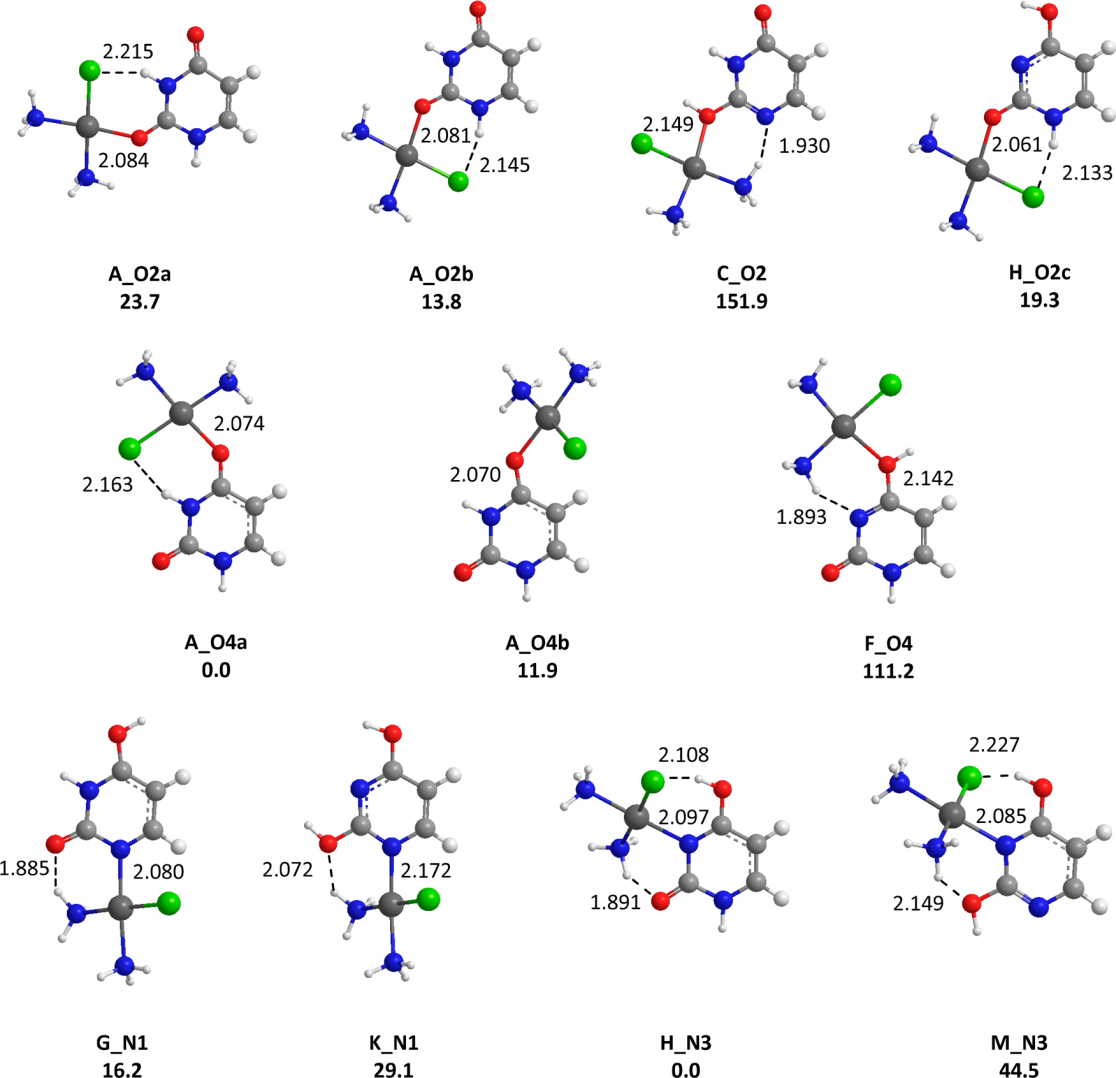
Representative structures obtained for the *cis*-[PtCl(NH_3_)_2_(U)]^+^ complexes. B3LYP/LACV3P/6-311G** relative free energies given in kJ/mol. Distances are given in Å.

**Scheme 1:**
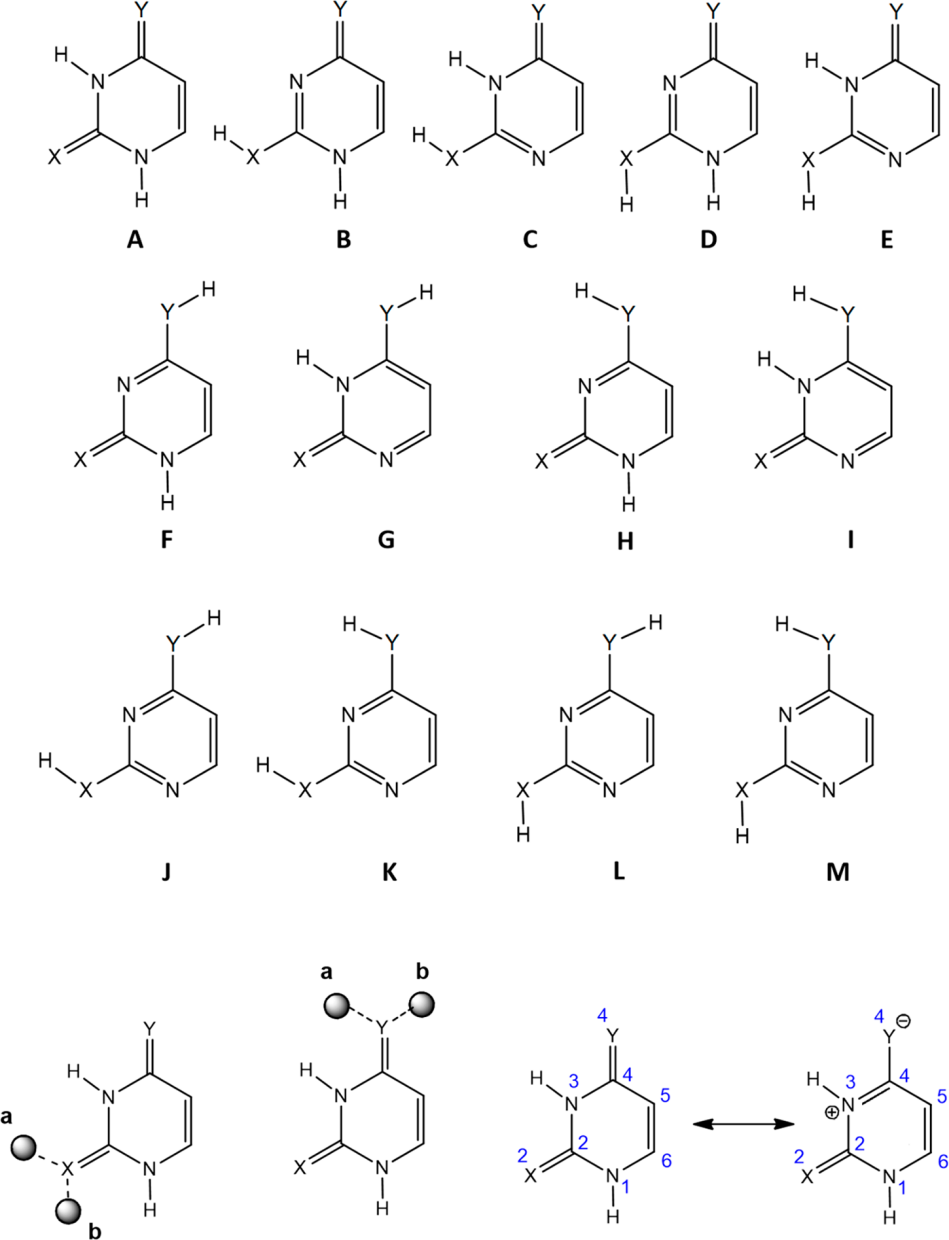
Nomenclature Adopted and Zwitterionic Effect for U (X = Y = O), 2SU (X = S, Y = O), 4SU (X = O, Y = S), and 24dSU (X = Y = S)

**Figure 4:**
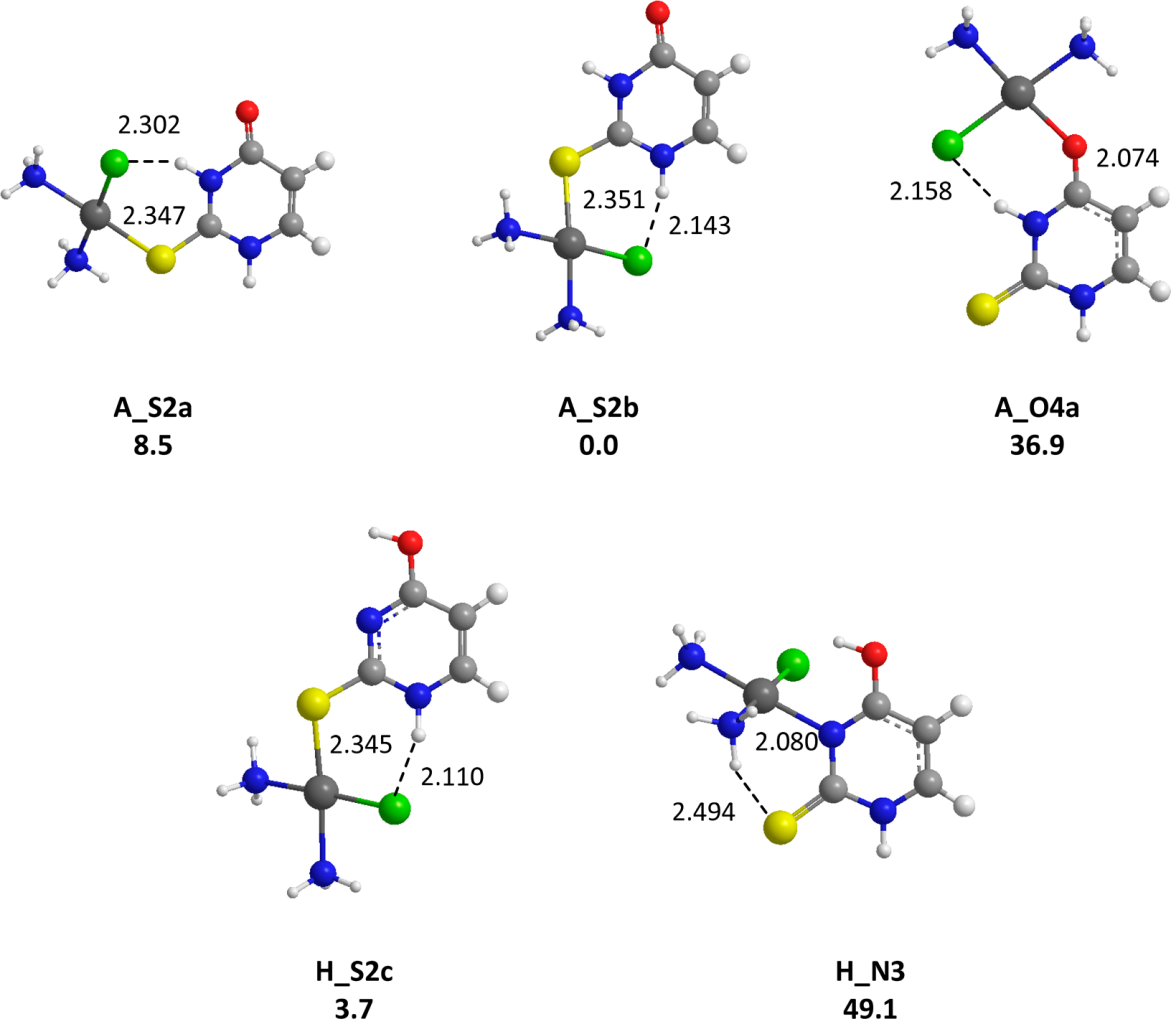
Representative structures obtained for the *cis*-[PtCl(NH_3_)_2_(2SU)]^+^ complexes. B3LYP/LACV3*P*/6-311G**relative free energies given in kJ/mol. Distances are given in Å.

**Figure 5:**
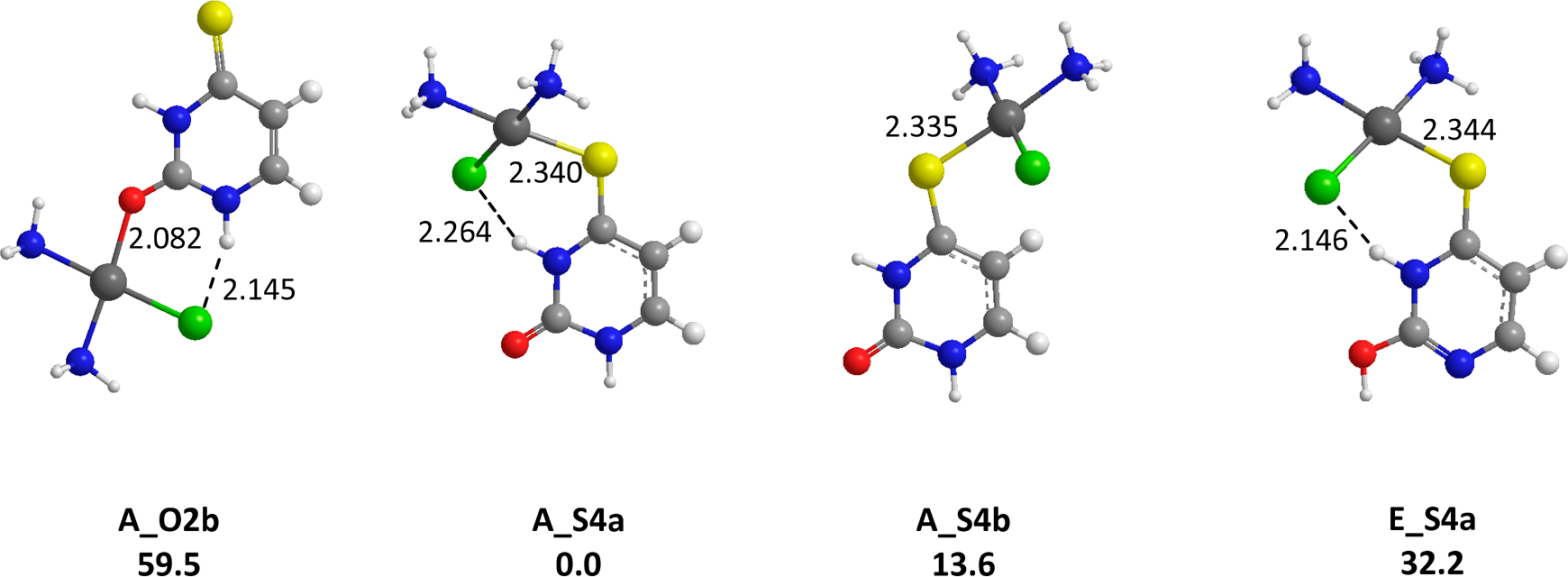
Representative structures obtained for the *cis*-[PtCl(NH_3_)_2_(4SU)]^+^ complexes. B3LYP/LACV3*P*/6-311G**relative free energies given in kJ/mol. Distances are given in Å.

**Figure 6:**
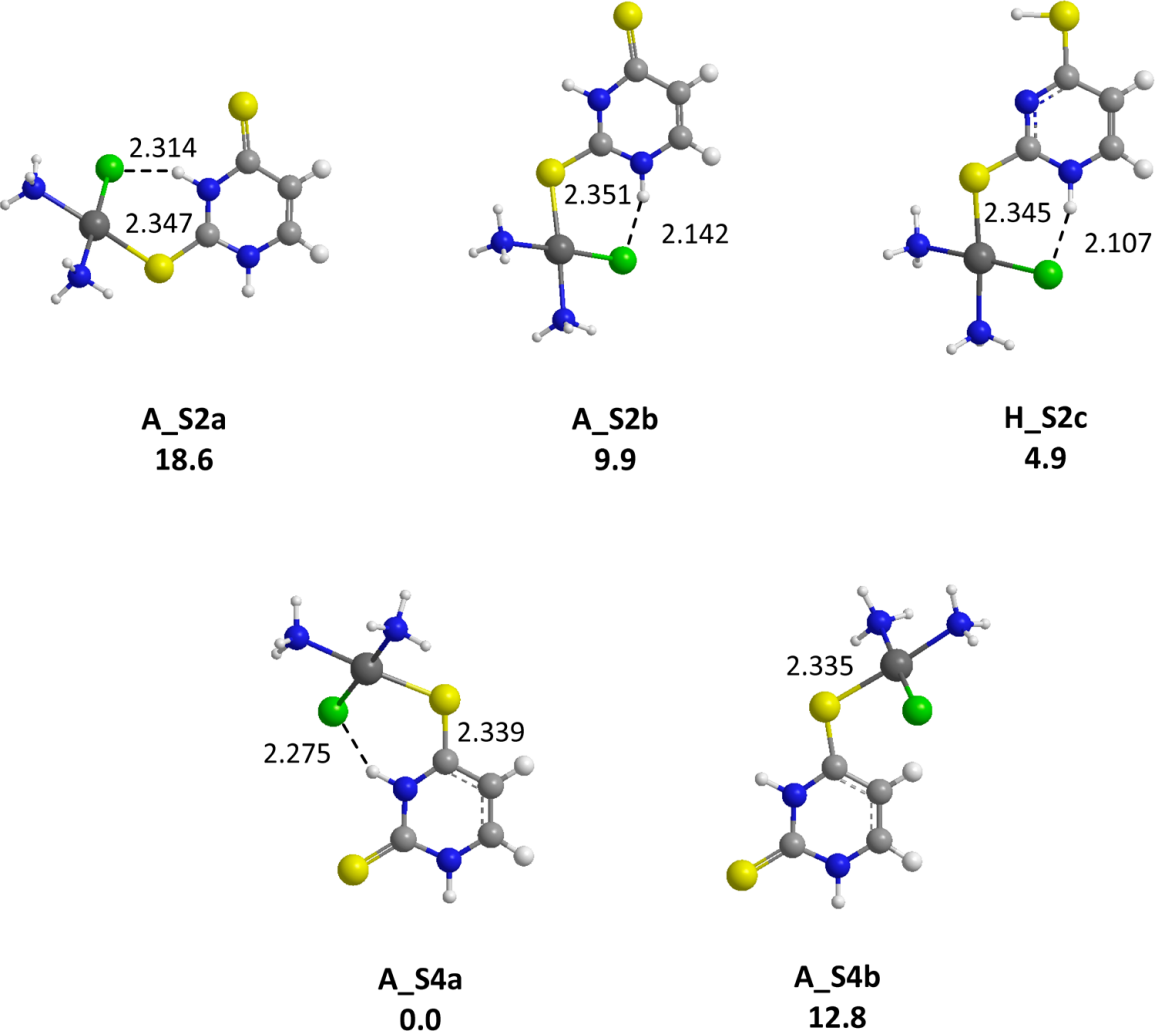
Representative structures obtained for the *cis*-[PtCl(NH_3_)_2_(24dSU)]^+^ complexes. B3LYP/LACV3P/6-311G**relative free energies given in kJ/mol. Distances are given in Å.

The potential energy surface for the four *cis*-[PtCl(NH_3_)_2_(L)]^+^ (L = U, 2SU, 4SU, 24dSU) complexes has been extensively explored in order to interpret their IRMPD spectra and gain a structural characterization. To this end, 13 different tautomers of uracil and thiouracils have been considered, whose optimized structures and corresponding relative free energies at 298 K (kJ mol^–1^) are given in Figures 3–6. Tables S1 and S2 in the Supporting Information provide comprehensive thermodynamic information on these and other identified structures deriving from canonical and alternative forms, while the whole set of optimized geometries are reported in Figures S11–S14 in the SI. In order to label the structures, the **T_Znm** nomenclature has been adopted, where **T** identifies the tautomer considered, as shown in Scheme 1, **Z** is the binding atom (O, N or S), **n** being the atom number, and **m** (m= a, b, c...) a letter which identifies the different conformers for a given coordination scheme. To be noted, for the canonical form of the nucleobase (**A**), the letter **a** (**b**) was systematically attributed to the complex in which the Pt atom is pointing toward (away from) the N3 center (Scheme 1).

If one first considers the uracil complexes, *cis*-[PtCl(NH_3_)_2_U]^+^, one may note that the most stable form, **A_O4a**, is characterized by *cis*-[PtCl(NH_3_)_2_]^+^ binding with the O4 keto center of uracil, the metal leaning toward N3 (Figure 3). The structure **A_O4b**, associated with the alternate orientation, is found to be ∼12 kJ/mol higher in energy, probably due to the loss of the hydrogen bond between the chlorine atom and the NH group. Examination of Figure 3 also shows that binding with O2 results in less stable forms. Concerning the canonical forms of uracil, this behavior has already been reported for complexation by alkali cations,^33,58,60^ Cu^+^ and Cu^2+^ ions,^27,70^ as well as Ca^2+^ and Pb^2+^,^29,30^ and may be attributed to the so-called “zwitterionic effect” introduced some years ago by Lamsabhi et al.^11^ In this study, it was shown through the analysis of the charge distribution of uracil and thiouracils that the contribution of zwitterionic resonance structures is important in this kind of interaction. This effect results in an increased negative charge on the heteroatom at position 4 (Y) with respect to the one attached to position 2 (X), and therefore in an increase of its intrinsic basicity toward the approaching Lewis acid. We also considered the possible coordination of the Pt center with enol groups (see for example **C_O2** or **F_O4** forms) of tautomeric forms. Such interactions result in significantly less stable structures, as attested by the computed relative free energies. On the other hand, several structures, all characterized by the occurrence of intramolecular hydrogen bonds involving the amino or chlorido ligands of the *cis*-[PtCl(NH_3_)_2_]^+^ moiety and an electronegative atom or NH group of the nucleobase, appear particularly stable. This is notably the case of the tautomeric forms **H_O2c** and **H_N3**, the latter lying at the same energy level as **A_O4a**. For Ca^2+^ and Cu^2+^ ions, theoretical studies have demonstrated that complexes involving tautomeric forms of uracil are much more stable than those involving canonical forms, the associated proton shift allowing bidentate interactions with O2 and N3 centers.^27,30^

Examination of the structures optimized with canonical 2-thiouracil (Figure 4) shows that the preferred coordination site also depends on the nature of the heteroatom involved in the interaction. Our results confirm that the Pt center displays a stronger affinity for sulfur than for oxygen, probably due to the greater polarizability of sulfur. This evidence has already been reported for “soft” ions like Cu^+^,^70^ whereas the association of “hard” alkali cations or Cu^2+^ ions with the oxygen atom is systematically favored when the systems present both types of basic centers, that is, a keto and a thioketo group.^27,33,60^ Unlike previous evidence on Pb^2+^ ions,^29^ in presence of the *cis*-[PtCl(NH_3_)_2_]^+^ species, the enhanced affinity for sulfur outcompetes the zwitterionic effect in the case of 2-thiouracil, leading to **A_S2** forms significantly more stable than **A_O4** structure, the latter geometry being now located 36.9 kJ/mol above the global minimum (**A_S2b**, Figure 4). We also optimized numerous structures involving tautomeric forms of 2SU. One form, namely **H_S2c** is found particularly stable, but remains slightly above the canonical **A_S2b** form (+3.7 kJ/mol). Within **H_S2c**, the coordination remains monodentate satisfying the square planar coordination of platinum(II), whereas bidentate interactions with tautomeric forms were reported as the most stable structures for Cu^2+^ and Ca^2+^ ions.^27,30^

In the case of 4-thiouracil, the zwitterionic effect and the bias for platinum binding to sulfur concur in favoring coordination to S4 (**A_S4a**, Figure 5), the relative free energy difference with the **A_O2b** isomer reaching now ∼60 kJ/mol. The binding scheme observed in presence of cisplatin therefore differs from the one reported for alkali cations,^33,60^ as well as for Cu^2+^ and Ca^2+^ ions^27,30^ (preferred interaction with O2). Unlike uracil and 2-thiouracil, competitive tautomeric forms were not identified, the most stable, **E_S4a**, being 32.2 kJ/mol above the global minimum **A_S4a**.

Finally, in the presence of 2,4-dithiouracil which, like uracil, has equal X and Y basic centers, the most favorable coordination scheme (**A_S4a**, Figure 6) is driven only by the zwitterionic effect, with relative free energies comparable to those computed for uracil (Figure 3), in agreement with previous evidence.^27−30,58−60^ Noteworthily, a very stable complex involving a tautomeric form, **H_S2c**, is located on the potential energy surface, which is similar to the stable tautomeric form obtained with 2-thiouracil (vide supra).

#### IRMPD Spectra of *cis*-[PtCl(NH_3_)_2_(L)]^+^ Complexes

According to the computational analysis that points to a coordination scheme governed by the distinct contribution of a zwitterionic effect and the strong cisPt affinity for sulfur, different types of structures are likely to be present in the sampled ion population, depending on the number of sulfur atoms. This notion is consistent with the experimental findings by IRMPD spectroscopy (Figure 2), both in the fingerprint and the X-H stretch regions.

#### *cis*-[PtCl(NH_3_)_2_(U)]^+^ Complex

**Figure 7:**
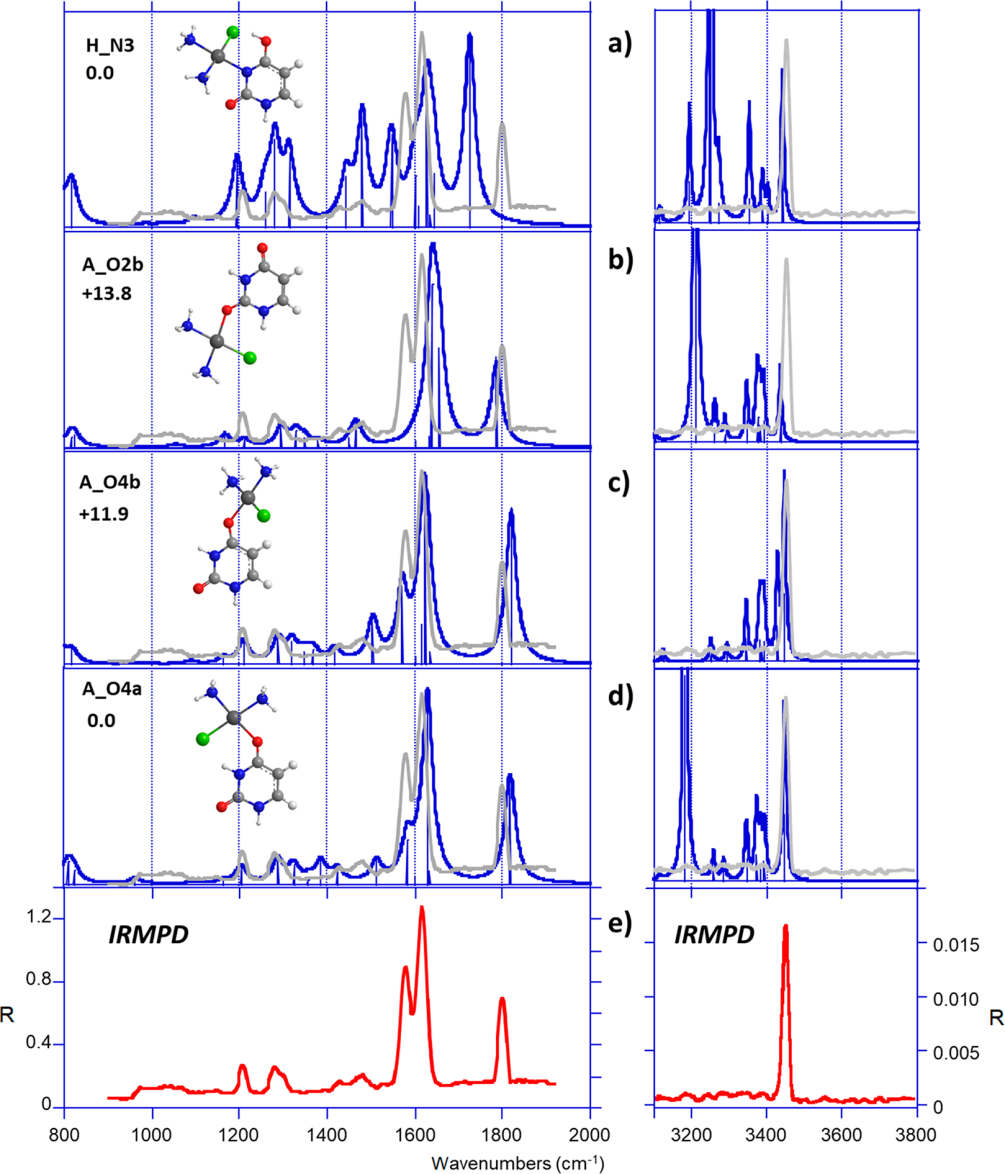
(e) IRMPD spectrum of *cis*-[PtCl(NH_3_)_2_(U)]^+^ compared with DFT-computed IR absorption spectra (a–d) of some relevant structures. The experimental IRMPD spectrum is overlaid in gray.

For this system, several structures are close in energy, two of them being degenerate (**A_O4a** and **H_N3**), thus suggesting that a mixture of tautomers might contribute to the sampled ion population. As a consequence, IRMPD spectroscopy can be particularly helpful to decipher the presence of multiple forms. In the fingerprint region, the IRMPD spectrum recorded for the *cis*-[PtCl(NH_3_)_2_(U)]^+^ complex has been compared with the DFT-computed vibrational spectra of low-lying structures (Figure 7). A very good agreement emerges between the experimental trace and the vibrational spectra computed for the O4-coordinated complexes (**A_O4a** and **A_O4b;**Figure 7c,d). The positions and intensities of the major IR active modes of these two forms are summarized in Table S3 of the Supporting Information. In particular, the IRMPD band detected at 1209 cm^–1^ can be interpreted as a combination of C–H and N1–H bending modes of uracil. The IRMPD signal detected at 1290 cm^–1^ can be assigned to the NH_3_′ umbrella bending mode (by convention the NH_3_′ group corresponds to the ammonia group in the *trans* position with respect to the chlorine atom). The weak bands measured at 1426 and 1480 cm^–1^ correspond to the N1H bending mode and the stretches of C4C5 and N1C6 bonds, respectively. The pronounced feature at 1580 cm^–1^ is ascribed to the C4O4 stretching mode, therefore significantly red-shifted as compared to an unperturbed C=O stretch, due to the interaction with Pt. As an additional argument, the C2=O2 stretch computed at 1817–1819 wavenumbers for both forms, **A_O4a** and **A_O4b**, well reproduces the experimental band at 1800 cm^–1^. The most intense signal, detected at 1615 cm^–1^, may be assigned to the C5C6 stretching mode. The **A_O2b** structure (Figure 7b) cannot be excluded on the basis of the spectroscopic features, although this structure does not correctly reproduce the experimental trace recorded between 1560 and 1620 cm^–1^. On the contrary, the **H_N3** form, even if degenerate with the global minimum, can be reasonably discarded (Figure 7a). Finally, in the X–H stretch region, only one particularly intense band was detected at 3450 cm^–1^. The signal is well reproduced by the N1–H stretching mode of both **A_O4a** and **A_O4b**. Vibrational modes associated with (strongly) hydrogen-bonded networks are barely observed in the experimental spectrum, including the intense N3–H stretch predicted for **A_O4a** at 3183 cm^–1^, and the weaker asymmetric NH_2_ stretches of NH_3_ and NH_3_′ in the 3370–3394 cm^–1^ and 3345–3392 cm^–1^ ranges for **A_O4a** and **A_O4b**, respectively. A similar behavior where H-bond interactions are not faithfully revealed has been already described as “IRMPD transparency” and reported for several (bio)molecular systems.^71−73^ To summarize, comparison between IRMPD data and DFT calculations demonstrate that the *cis*-[Pt(NH_3_)_2_Cl(U)]^+^ complex is mainly characterized by coordination of the cisPt moiety with O4 of the canonical form of uracil.

#### *cis*-[PtCl(NH_3_)_2_(2SU)]^+^ Complex

**Figure 8:**
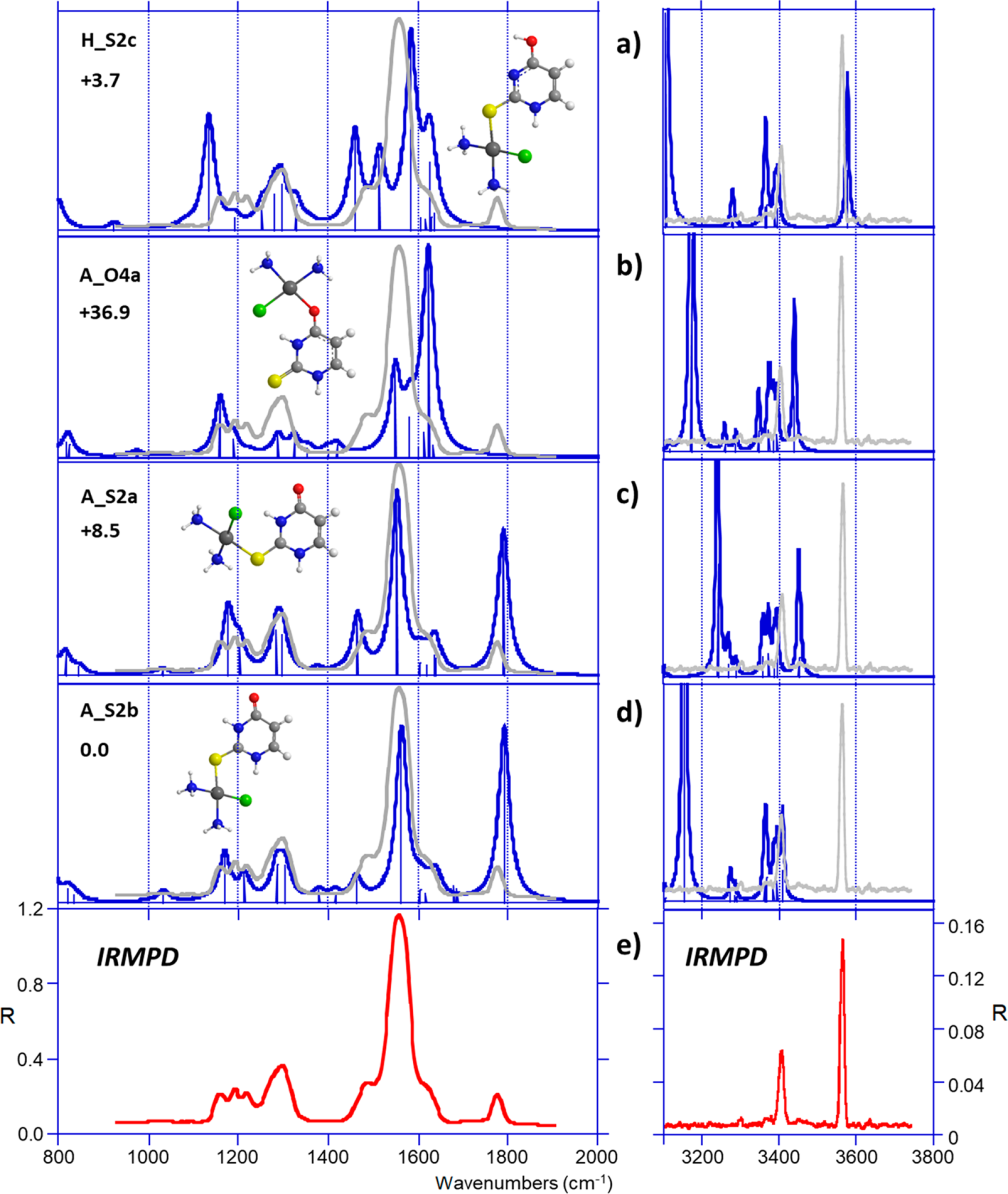
(e) IRMPD spectrum of *cis*-[PtCl(NH_3_)_2_(2SU)]^+^ compared with DFT-computed IR absorption spectra (a–d) of some relevant structures. The experimental IRMPD spectrum is overlaid in gray.

As illustrated in Figure 2, the experimental IRMPD spectrum of *cis*-[PtCl(NH_3_)_2_(2SU)]^+^ differs significantly from the one of the uracil complex. The comparison shown in Figure 8 indicates a very good agreement with the vibrational spectra computed for the global minimum, **A_S2b** and its rotamer **A_S2a**. Notably, both structures correctly reproduce the shape and the intensity of the experimental profile recorded between 1450 and 1650 cm^–1^, due to three bands attributed to N1C2 bond stretch, NH bending and NH_2_ scissoring modes of both NH_3_ and NH_3_′ groups, and to C5C6 bond stretch (Table S4). Other signals are also well reproduced. The band at 1770 cm^–1^ is attributed to the stretch of the C4=O4 carbonyl group computed at 1790 cm^–1^. The broad band at 1297 cm^–1^ corresponds to the umbrella modes of NH_3_′ and NH_3_, whereas series of unresolved features between 1145 and 1240 cm^–1^ may be ascribed to CH bending modes coupled with C2S2 stretch. In the X–H stretching region, the IRMPD spectrum is also different from that recorded with uracil as it exhibits two peaks, detected at 3405 and 3569 cm^–1^. Remarkably, the latter signal cannot be interpreted by vibrations of the S2-coordinated tautomer A, but rather suggests the presence of a certain fraction of **H_S2c**, tautomer of **A_S2b**, which lies only 3.7 kJ/mol above the global minimum and presents a free O4H stretch calculated at 3578 cm^–1^. Its vibrational spectrum also matches most of the bands observed in the fingerprint region, albeit with less overall agreement. Consequently, IRMPD data for 2SU seem to point to a mixture of tautomeric forms sharing the same coordination scheme (monodentate interaction with S2). Accordingly, formation of tautomeric forms have also been reported recently for protonated 2SU.^15^

#### *cis*-[PtCl(NH_3_)_2_(4SU)]^+^ Complex

**Figure 9:**
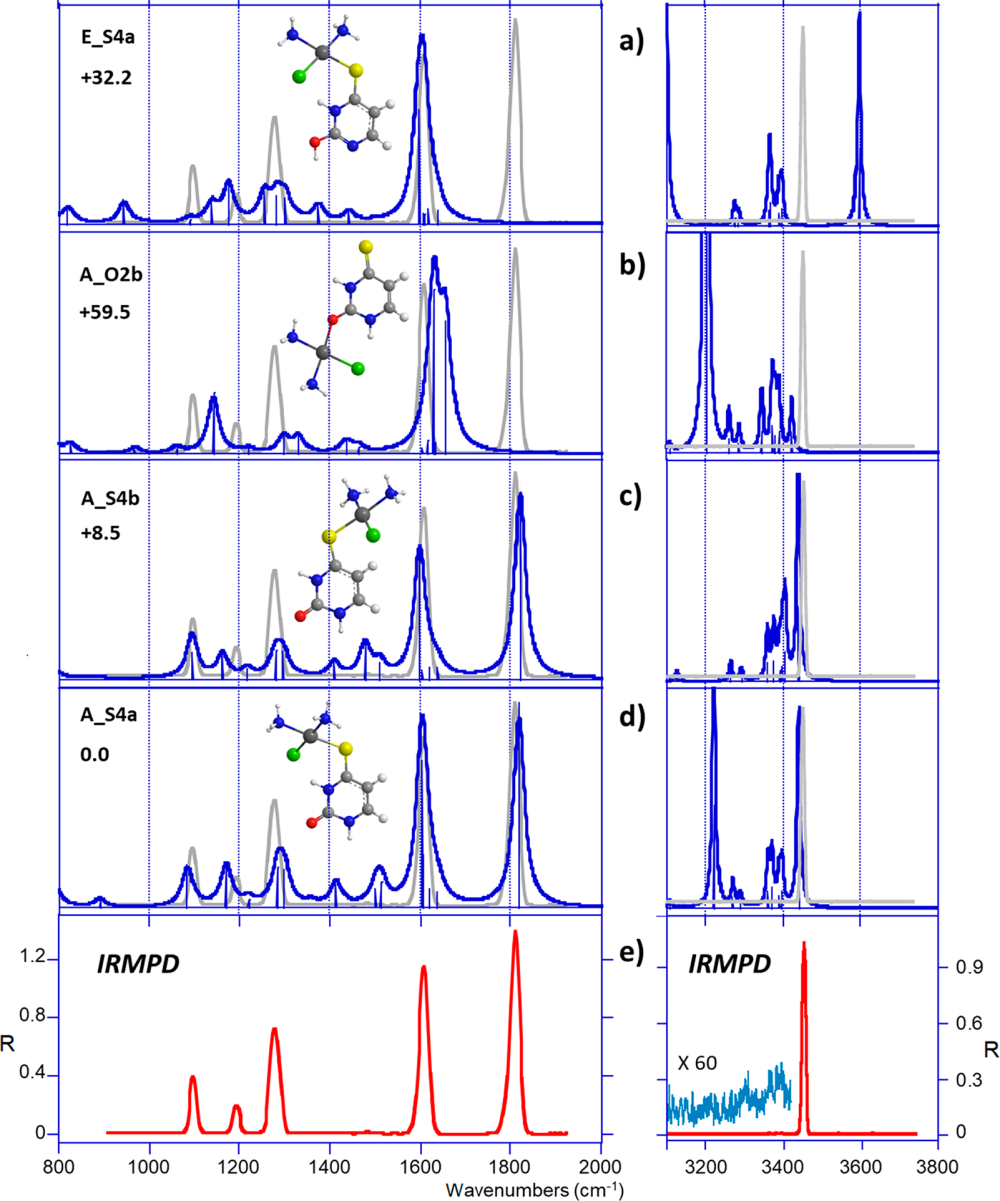
(e) IRMPD spectrum of *cis*-[PtCl(NH_3_)_2_(4SU)]^+^ compared with DFT-computed IR absorption spectra (a–d) of some relevant structures. The experimental IRMPD spectrum is overlaid in gray.

Again, the situation changes drastically when considering 4-thiouracil, thus allowing the two isomers of thiouracil complexes to be easily distinguished by IRMPD spectroscopy. As shown in Figure 9e, the experimental IRMPD spectrum in the fingerprint region exhibits five sharp signals with an unperturbed carbonyl group at 1808 cm^–1^, indicating a structure where the C2=O2 group does not interact with the metal. Consistently, the spectra computed for the **A_S4a** and **A_S4b** rotamers (Figure 9c,d) perfectly reproduce all the experimental bands. By order of increasing wavenumber, the three peaks at 1098, 1279, and 1609 cm^–1^ can be ascribed to C4S4 and C2N3 stretches, NH_3_ and NH_3_′ umbrella, and C5C6 stretch mode (Table S5). Formation of an O2-coordinated complex, lacking the carbonyl stretch, is unlikely because of a poorer agreement (Figure 9b), as expected from a structure which is already 60 kJ/mol higher in energy (vide supra). Finally, the tautomeric form **E_S4a** can also be discarded as it cannot account for any of the two absorptions observed at 1808 and 1098 cm^–1^. In the X-H stretch region, this form should give a strong band at about 3600 cm^–1^, due to the free O2H stretch, which is missing in the IRMPD spectrum, and cannot account for the only signal observed experimentally at 3452 cm^–1^. Such a strong single peak, computed for both **A_S4a** and **A-S4b**, is due to the free N1H stretch. As a consequence, our data suggest that a single type of structure is generated in presence of 4-thiouracil, characterized by the complexation of the cisPt moiety onto the sulfur atom of the canonical form. This was quite expected based on the computational survey where additive effects predict a favored attack at S4.

#### *cis*-[PtCl(NH_3_)_2_(24dSU)]^+^ Complex

**Figure 10:**
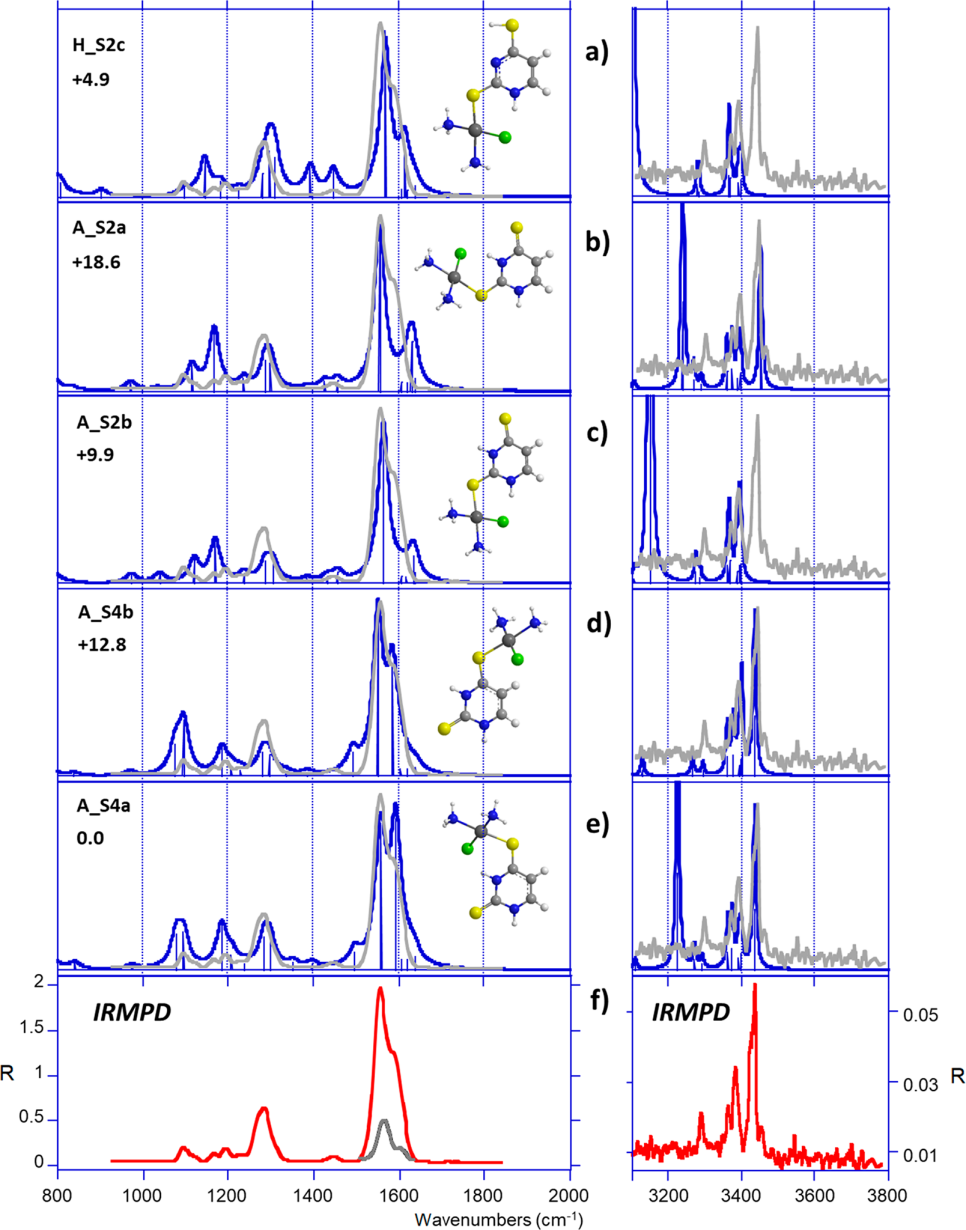
(f) IRMPD spectrum of *cis*-[PtCl(NH_3_)_2_(24dSU)]^+^ compared with DFT-computed IR absorption spectra (a–e) of some relevant structures. The experimental IRMPD spectrum is overlaid in gray.

Figure 10f shows the experimental IRMPD spectrum compared with the IR spectra computed for low-lying S4- and S2-coordinated complexes (Figure 10a–e). Notably, the best agreement is obtained with the S4 forms, well accounting for the feature recorded above 1500 cm^–1^, which can be interpreted as the combination of C2S2 and C5C6 stretches, computed at 1556 and 1591 cm^–1^, respectively (Table S6). The three experimental signals detected below 1400 cm^–1^ are also well reproduced by both **A_S4a** and **A_S4b**, although this finding is also true for the other structures. Remarkably, **H_S2c** and **A_S2b** isomers are the only ones that reproduce the weak experimental band at 1445 cm^–1^, suggesting that a minor contribution of these species may likely occur. This is a reasonable assumption as a previous IRMPD study has shown that sodium cationization promotes the stabilization of a minor tautomer of 24dSU.^32^

Examination of the IRMPD spectrum recorded in the OPO range shows that the three experimental signals detected at 3285, 3380, and 3431 cm^–1^ are well reproduced by the vibrational spectra of **A_S4a** and **A_S4b**, corresponding to the symmetric and asymmetric stretches of the ammonia ligands, and free N1H stretch, respectively. The N3H stretch computed at 3400 cm^–1^ in the case of **A_S4b**, is red-shifted at 3224 cm^–1^ for **A_S4a** due to the hydrogen bond established with the chlorine atom. According to previous discussion, this particular signal is barely observed experimentally. Finally, the weak shoulder detected at 3452 cm^–1^ is reproduced neither by the couple **A_S4a**/**A_S4b** nor by the tautomeric form **H_S2c**, thus suggesting that some amount of an additional structure could be present. This form might correspond to a S2-coordinated complex, as the N1H stretch computed at 3451 cm^–1^ for **A_S2a** satisfyingly matches with the experimental band (3452 cm^–1^). In summary, the interaction of cisPt with 24dSU likely originates a mixture of different forms, the canonical S4-coordinated complexes being largely the most populated.

## Conclusions

The interaction between the antitumor drug cisplatin and four (thio)uracils in aqueous methanol solution yields monofunctional primary complexes *cis*-[PtCl(NH_3_)_2_(L)]^+^ that were extracted by ESI and delivered to the gas phase as isolated species to be characterized by IR spectroscopy in the complementary hydrogen-stretching and fingerprint ranges. The prevailing dissociation channel observed upon both CID and IRMPD assay, involving loss of NH_3_, is consistent with the dominant process previously reported for similar cisplatin adducts.^37,38,40−42^

The experimentally accessed structures have been identified by comparison with the linear IR spectra of candidate structures generated by quantum chemical calculations.

The ground-state structures of *cis*-[PtCl(NH_3_)_2_(L)]^+^ complexes have shown sulfur as the preferred complexation site. The most favored binding of platinum is directed to the O4(S4) atoms of the canonical forms of U, 4SU and 24dSU, and to the S2 atom of 2SU. However, the present results suggest that, while *cis*-[PtCl(NH_3_)_2_(L)]^+^ (L = U, 4SU) are represented by a single canonical structure, platinum(II) cationization stabilizes also minor, noncanonical tautomers for L = 2SU and 24dSU. Therefore, although to a much more limited extent relative to protonation, also the *cis*-[PtCl(NH_3_)_2_]^+^ subunit of cisplatin shows an augmented prospect of nucleic acid strand disorder.

## Supporting Information Available

CID mass spectra (Figures S1–S4); breakdown curves (Figures S4–S6); ESI mass spectra after irradiation with CLIO FEL light (Figures S7–S10); optimized geometries and relative free energy values (Figures S11–S14); thermodynamic data (Tables S1–S2); experimental and computed IR bands (Tables S3–S6) (PDF)
