# Supplementary material for: Insights into Cisplatin Binding to Uracil and Thiouracils from IRMPD Spectroscopy and Tandem Mass Spectrometry
Source: J Am Soc Mass Spectrom. 2020 Feb 18;31(4):946–60. doi: 10.1021/jasms.0c00006 (PMC7997577; doi:10.1021/jasms.0c00006)
Supplement: Supplementary file 1 — js0c00006_si_001.pdf [file js0c00006_si_001.pdf]

Supporting Information for:

Insights into Cisplatin Binding to Uracil and Thiouracils from IRMPD Spectroscopy and Tandem Mass Spectrometry

Davide Corinti[1,] Maria Elisa Crestoni*[1], Barbara Chiavarino[1], Simonetta Fornarini[1], Debora Scuderi,[2] and Jean-Yves Salpin*[3,4]

1) Dipartimento di Chimica e Tecnologie del Farmaco, Università di Roma "La Sapienza", P.le A. Moro 5, 00185 Roma, ITALY

2) Université Paris-Saclay, CNRS, Institut de Chimie Physique UMR8000, 91405, Orsay, France

3) Université Paris-Saclay, CNRS, Univ Evry, LAMBE, Evry-Courcouronnes, 91025, France

4) CY Cergy Paris Université, LAMBE, Evry-Courcouronnes, 91025, France

Corresponding Authors:

mariaelisa.crestoni@uniroma1.it (M.E.C.)

jeanyves.salpin@univ-evry.fr (J.-Y.S.)

Contents:

Computational Details. Any additional computational detail and material (xyz files of computed structures, calculated vibrational frequencies for all structures, etc.) is available from the authors upon request.

Figure S1. CID mass spectrum recorded upon activation of the *cis*-$[PtCl(NH_3)_2(2SU)]^+$ ion (*m/z* 391) using a collision energy of 5 eV.

Figure S2. CID mass spectrum recorded upon activation of the *cis*-$[PtCl(NH_3)_2(4SU)]^+$ ion (*m/z* 391) using a collision energy of 5 eV.

Figure S3. CID mass spectrum recorded upon activation of the *cis*-$[PtCl(NH_3)_2(24SU)]^+$ ion (*m/z* 407) using a collision energy of 5 eV.

Figure S4. Breakdown curves for *cis*-$[PtCl(NH_3)_2(2SU)]^+$. The red profile reports the precursor ion decay, while the blue profile shows the increasing abundance of the sum of all the product ions. The retarding potential experiment performed to obtain the corrected ECM is presented in the bottom panel.

Figure S5. Breakdown curves for *cis*-$[PtCl(NH_3)_2(4SU)]^+$. The red profile reports the precursor ion decay, while the blue profile shows the increasing abundance of the sum of all the product ions. The retarding potential experiment performed to obtain the corrected ECM is presented in the bottom panel.

Figure S6. Breakdown curves for *cis*-$[PtCl(NH_3)_2(24SU)]^+$. The red profile reports the precursor ion decay, while the blue profile shows the increasing abundance of the sum of all the product ions. The retarding potential experiment performed to obtain the corrected ECM is presented in the bottom panel.

Figure S7. Positive ESI mass spectra recorded upon selection of *cis*-$[PtCl(NH_3)_2(U)]^+$ ion (*m/z* 375-379), in a hybrid FT-ICR tandem mass spectrometer (APEX-Qe Bruker Daltonics) after irradiation with CLIO FEL light on resonance at 1800 cm^{-1}.

Figure S8. Positive ESI mass spectra recorded upon selection of *cis*-$[PtCl(NH_3)_2(2SU)]^+$ ion (*m/z* 391-395), in a hybrid FT-ICR tandem mass spectrometer (APEX-Qe Bruker Daltonics) after irradiation with CLIO FEL light on resonance at 1488 cm^{-1}.

Figure S9. Positive ESI mass spectra recorded upon selection of *cis*-$[PtCl(NH_3)_2(4SU)]^+$ ion (*m/z* 391-395), in a hybrid FT-ICR tandem mass spectrometer (APEX-Qe Bruker Daltonics) after irradiation with CLIO FEL light on resonance at 1280 cm^{-1}.

Figure S10. Positive ESI mass spectra recorded upon selection of *cis*-$[PtCl(NH_3)_2(24dSU)]^+$ ion (*m/z* 407-411), in a hybrid FT-ICR tandem mass spectrometer (APEX-Qe Bruker Daltonics) after irradiation with CLIO FEL light on resonance at 1285 cm^{-1}.

Figure S11. Optimized geometries and relative free energy values (at the B3LYP/LACV3P/6-311G** level) at 298 K (kJ mol^{-1}) of *cis*-$[PtCl(NH_3)_2(U)]^+$. Noncovalent interactions are marked by dashed lines. Distances are given in Å.

Figure S12. Optimized geometries and relative free energy values (at the B3LYP/LACV3P/6-311G** level) at 298 K (kJ mol^{-1}) of *cis*-$[PtCl(NH_3)_2(2SU)]^+$. Noncovalent interactions are marked by dashed lines. Distances are given in Å.

Figure S13. Optimized geometries and relative free energy values (at the B3LYP/LACV3P/6-311G** level) at 298 K (kJ mol^{-1}) of *cis*-$[PtCl(NH_3)_2(4SU)]^+$. Noncovalent interactions are marked by dashed lines. Distances are given in Å.

Figure S14. Optimized geometries and relative free energy values (at the B3LYP/LACV3P/6-311G** level) at 298 K (kJ mol^{-1}) of *cis*-$[PtCl(NH_3)_2(24dSU)]^+$. Noncovalent interactions are marked by dashed lines. Distances are given in Å.

Table S1. Thermodynamic data for the lowest energy conformers and isomers of *cis*-$[PtCl(NH_3)_2(U)]^+$ calculated at the B3LYP/LACV3P/6-311G** level of theory.

Table S2. Thermodynamic data for the lowest energy conformers and isomers of *cis*-$[PtCl(NH_3)_2(L)]^+$ (L=2SU, 4SU, 24dSU) calculated at the B3LYP/LACV3P/6-311G** level of theory.

Table S3. Experimental and computed IR vibrational bands for *cis*-$[PtCl(NH_3)_2(U)]^+$.

Table S4. Experimental and computed IR vibrational bands for *cis*-$[PtCl(NH_3)_2(2SU)]^+$.

Table S5. Experimental and computed IR vibrational bands for *cis*-$[PtCl(NH_3)_2(4SU)]^+$.

Table S6. Experimental and computed IR vibrational bands for *cis*-$[PtCl(NH_3)_2(24dSU)]^+$.

Reference for Gaussian 09

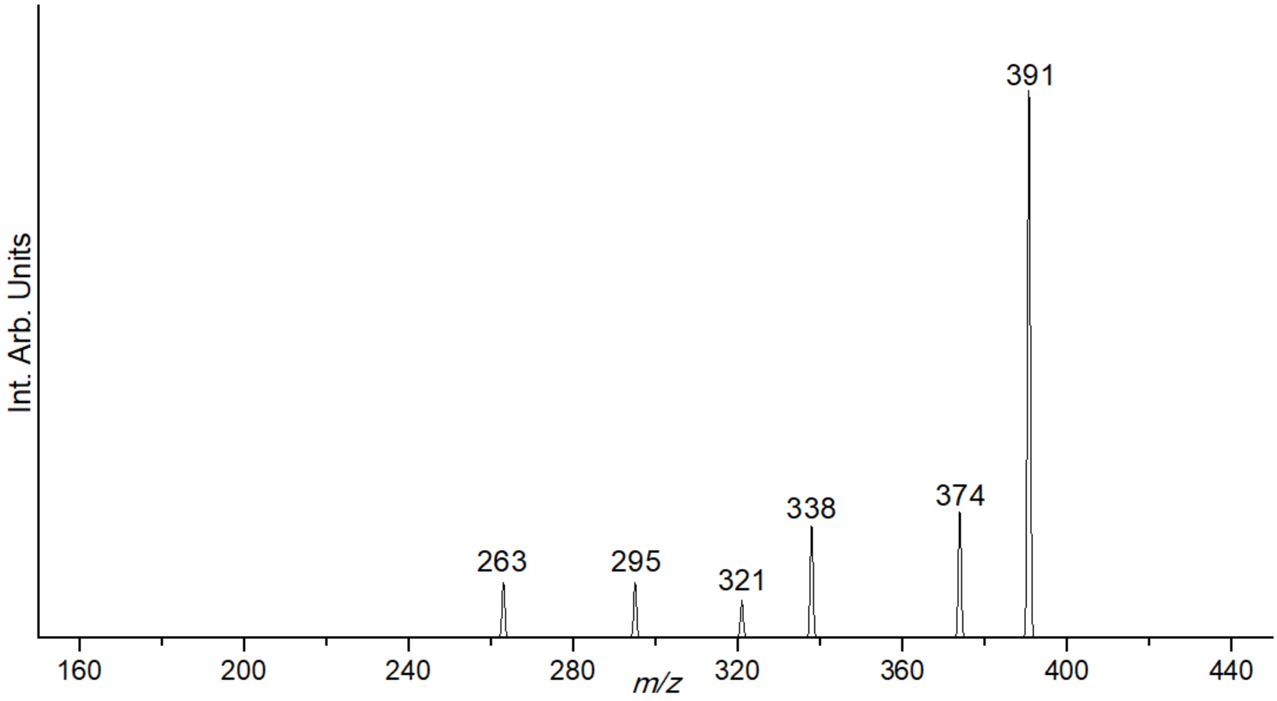

Figure S1. CID mass spectrum recorded upon activation of the *cis*-$[PtCl(NH_3)_2(2SU)]^+$ ion (*m/z* 391) using a collision energy of 5 eV.

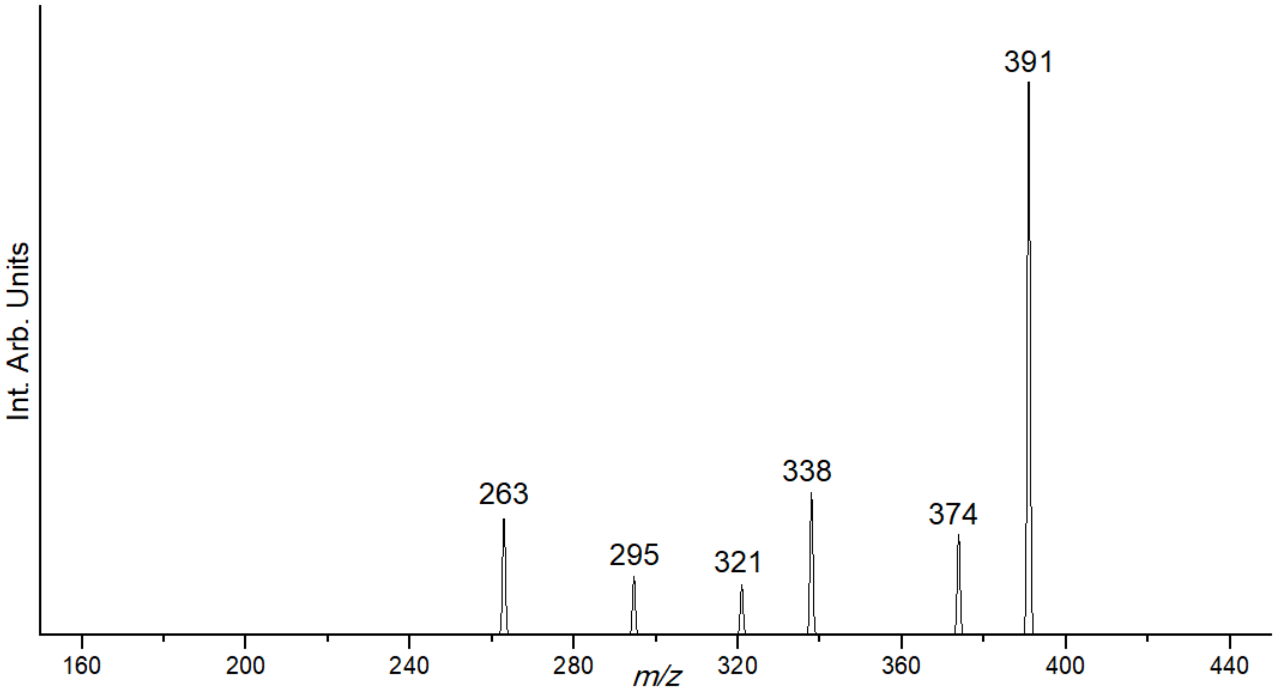

Figure S2. CID mass spectrum recorded upon activation of the *cis*-$[PtCl(NH_3)_2(4SU)]^+$ ion (*m/z* 391) using a collision energy of 5 eV.

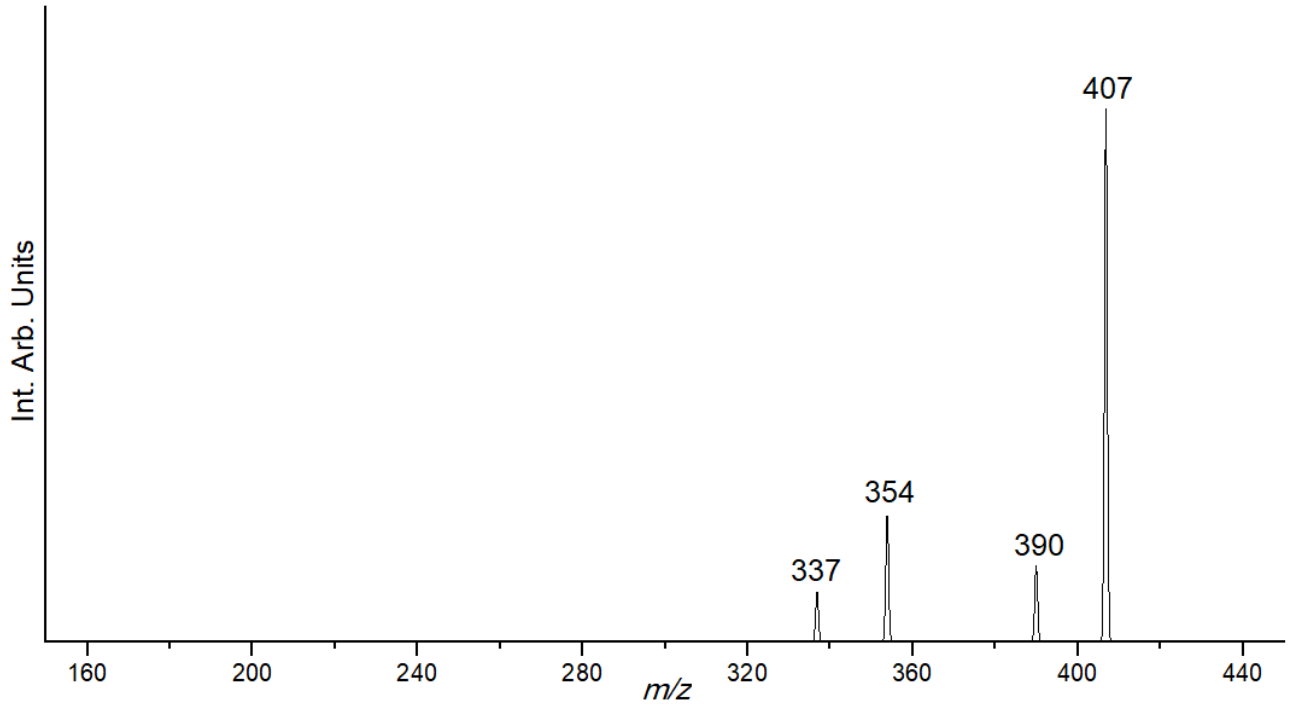

Figure S3. CID mass spectrum recorded upon activation of the *cis*-$[PtCl(NH_3)_2(24SU)]^+$ ion (*m/z* 407) using a collision energy of 5 eV.

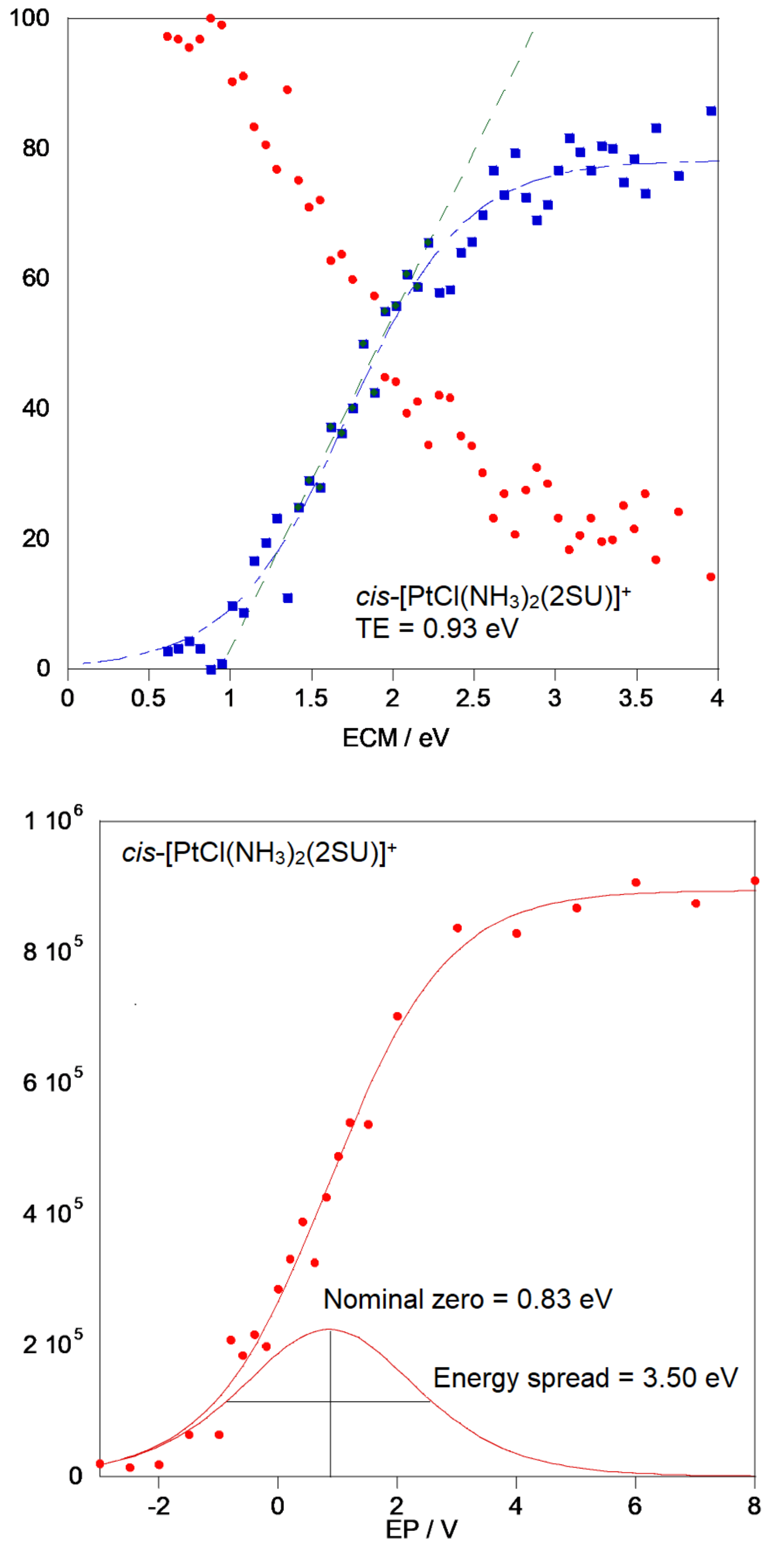

Figure S4. Breakdown curves for *cis*-[PtCl(NH_3)$_2$(2SU)]$^+$. The red profile reports the precursor ion decay, while the blue profile shows the increasing abundance of the sum of all the product ions. The retarding potential experiment performed to obtain the corrected E_{CM} is presented in the bottom panel.

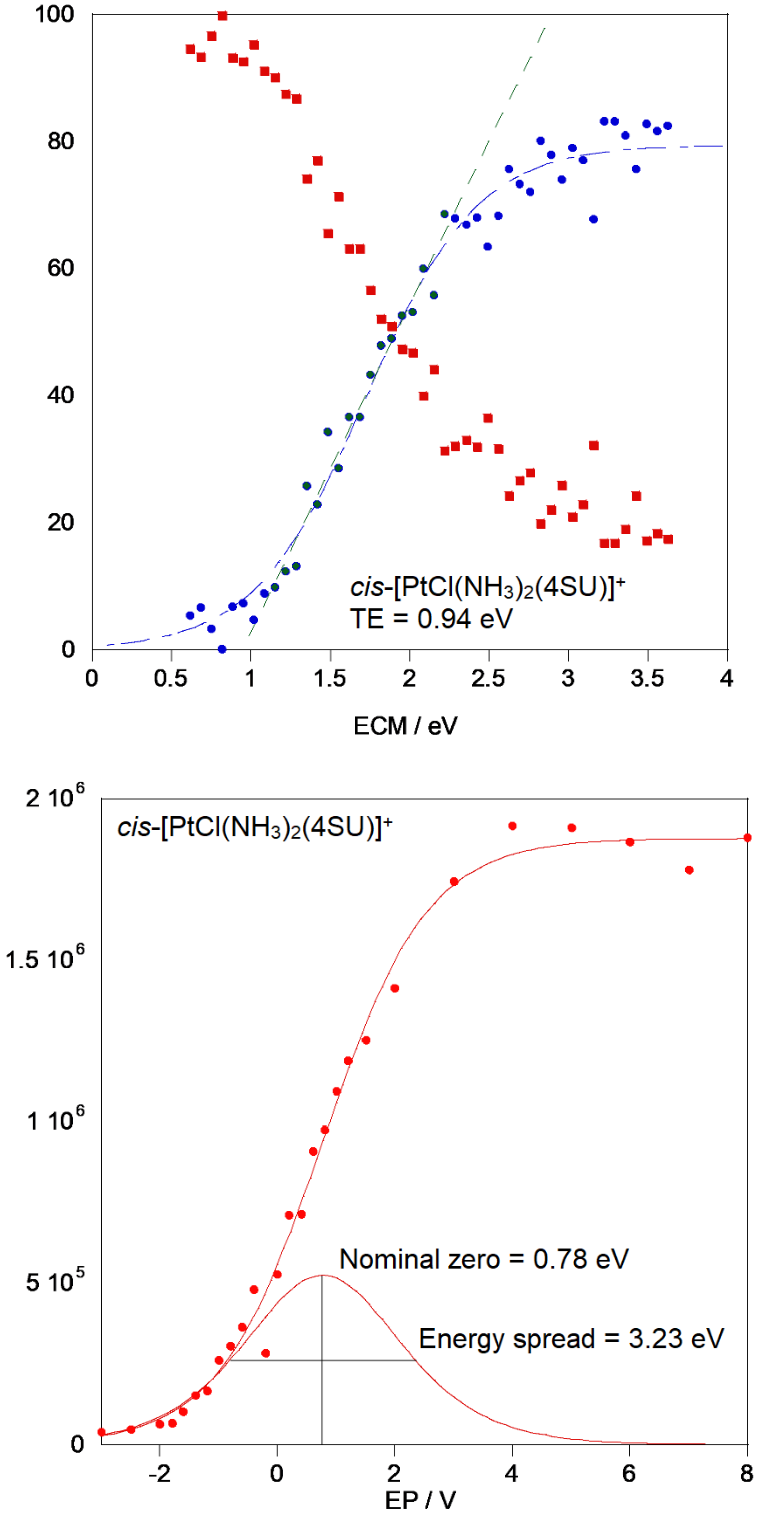

Figure S5. Breakdown curves for *cis*-$[PtCl(NH_3)_2(4SU)]^+$. The red profile reports the precursor ion decay, while the blue profile shows the increasing abundance of the sum of all the product ions. The retarding potential experiment performed to obtain the corrected E_{CM} is presented in the bottom panel.

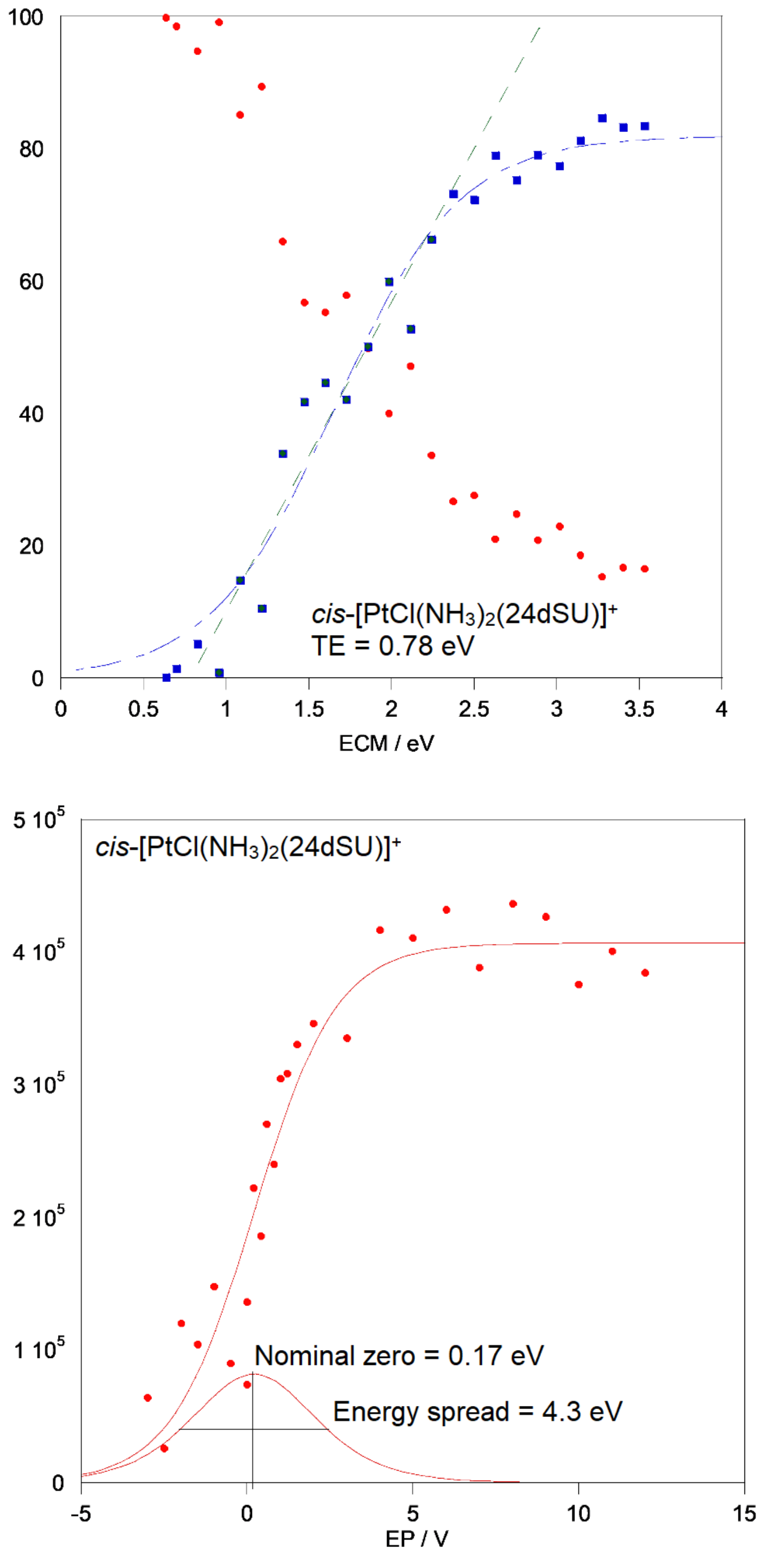

Figure S6. Breakdown curves for *cis*-$[PtCl(NH_3)_2(24dSU)]^+$. The red profile reports the precursor ion decay, while the blue profile shows the increasing abundance of the sum of all the product ions. The retarding potential experiment performed to obtain the corrected E_{CM} is presented in the bottom panel.

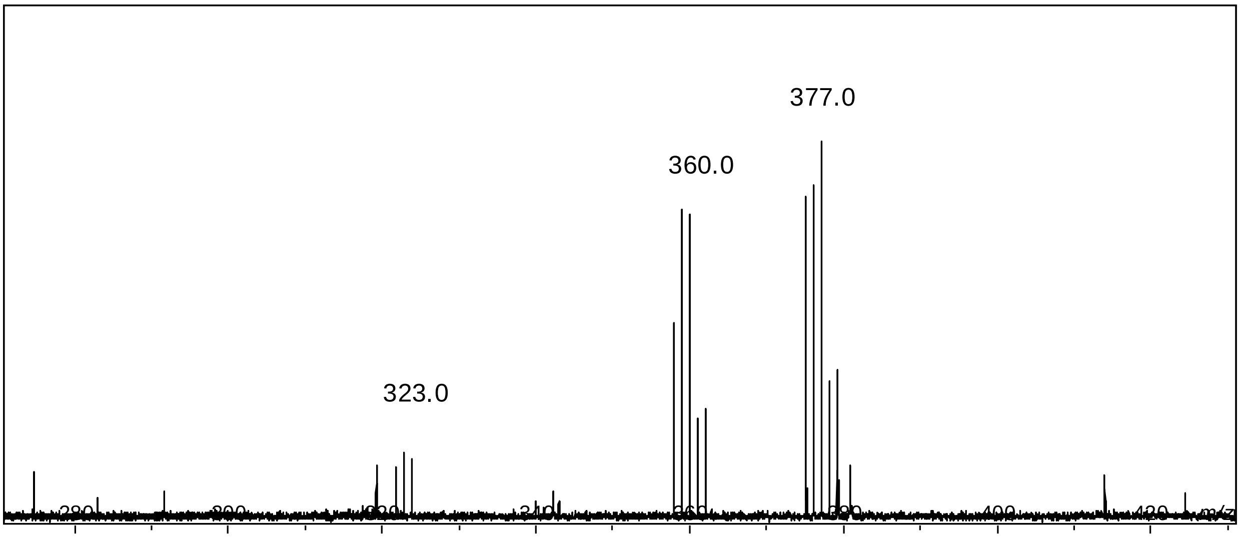

Figure S7. Positive ESI mass spectrum recorded upon selection of *cis*-[PtCl(NH_3)$_2$(U)]$^+$ ion (*m/z* 375-379), in a hybrid FT-ICR tandem mass spectrometer (APEX-Qe Bruker Daltonics) after irradiation with CLIO FEL light on resonance at 1800 cm^{-1}.

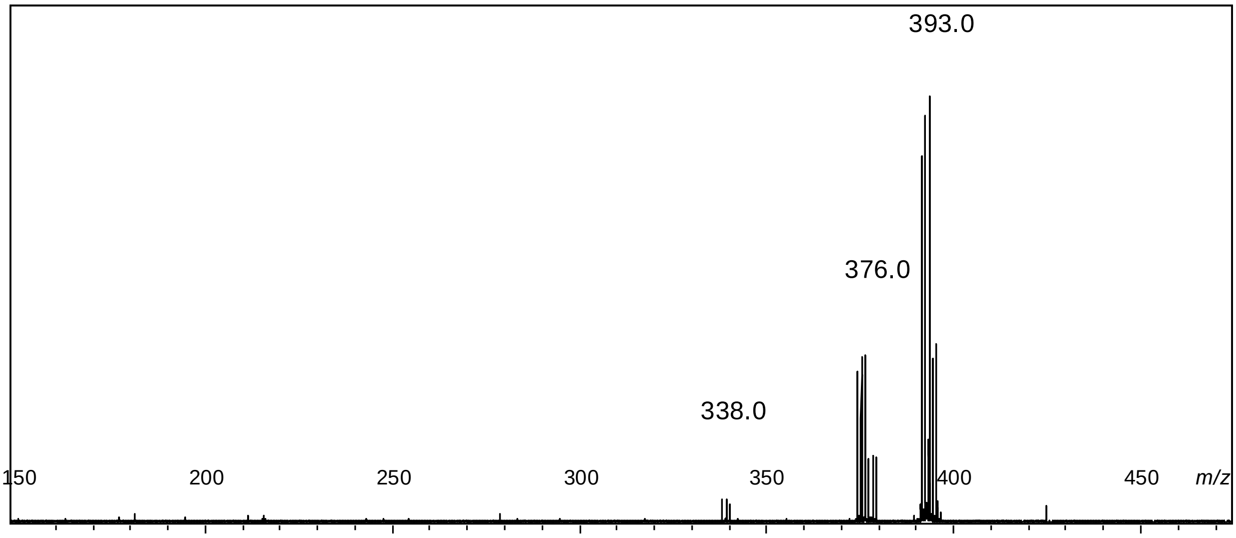

Figure S8. Positive ESI mass spectrum recorded upon selection of *cis*-[PtCl(NH_3)$_2$(2SU)]$^+$ ion (*m/z* 391-395), in a hybrid FT-ICR tandem mass spectrometer (APEX-Qe Bruker Daltonics) after irradiation with CLIO FEL light on resonance at 1488 cm^{-1}.

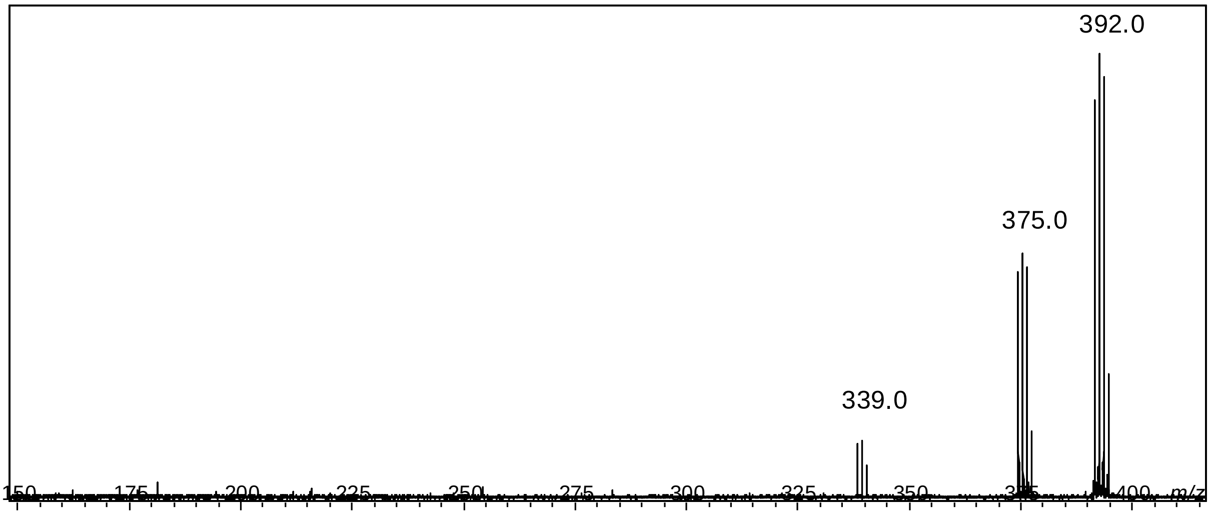

Figure S9. Positive ESI mass spectrum recorded upon selection of *cis*-[PtCl(NH$_3$)$_2$(4SU)]$^+$ ion (*m/z* 391-395), in a hybrid FT-ICR tandem mass spectrometer (APEX-Qe Bruker Daltonics) after irradiation with CLIO FEL light on resonance at 1280 cm^{-1}.

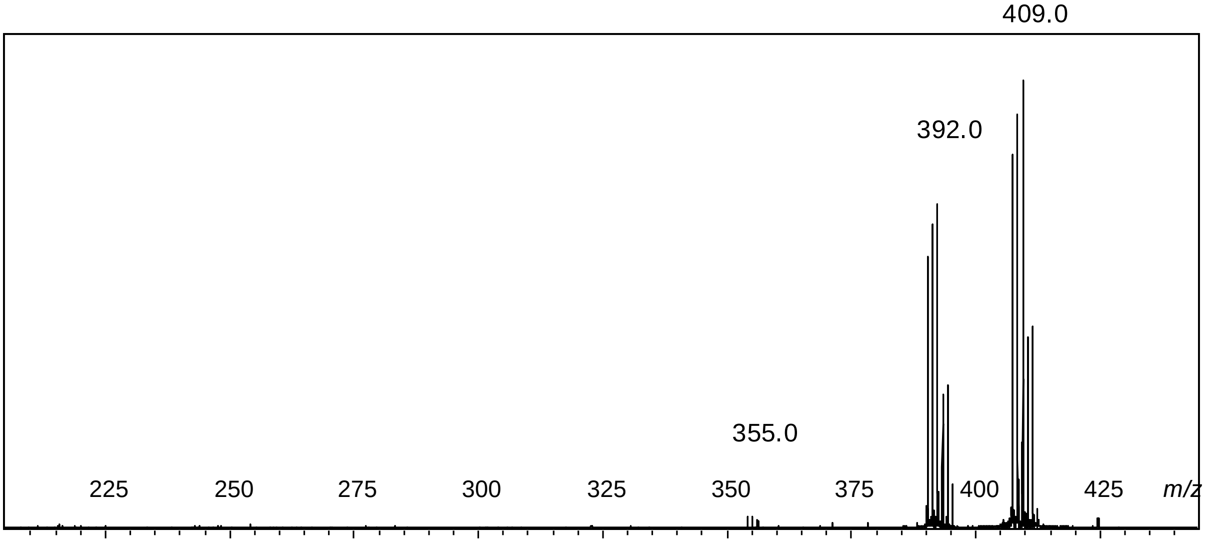

Figure S10. Positive ESI mass spectrum recorded upon selection of *cis*-[PtCl(NH$_3$)$_2$(24dSU)]$^+$ ion (*m/z* 407-411), in a hybrid FT-ICR tandem mass spectrometer (APEX-Qe Bruker Daltonics) after irradiation with CLIO FEL light on resonance at 1285 cm^{-1}.

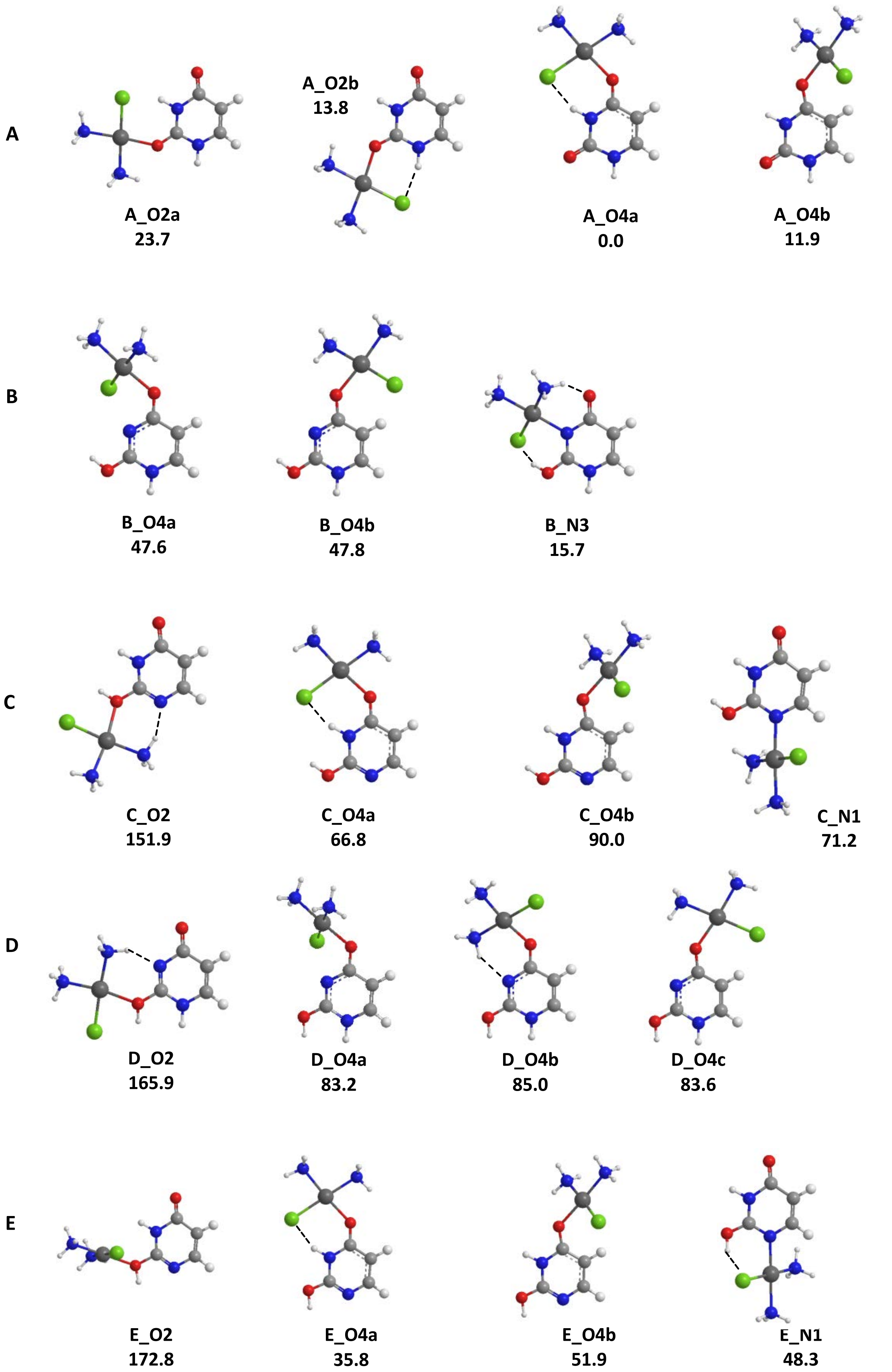

Figure S11a. Optimized geometries and relative free energy values (at the B3LYP/LACV3P/6-311G** level) at 298 K (kJ mol^{-1}) of *cis*-$[PtCl(NH_3)_2(U)]^+$ conformer and isomer families A, B, C, D, E. Noncovalent interactions are marked by dashed lines. Distances are given in Å.

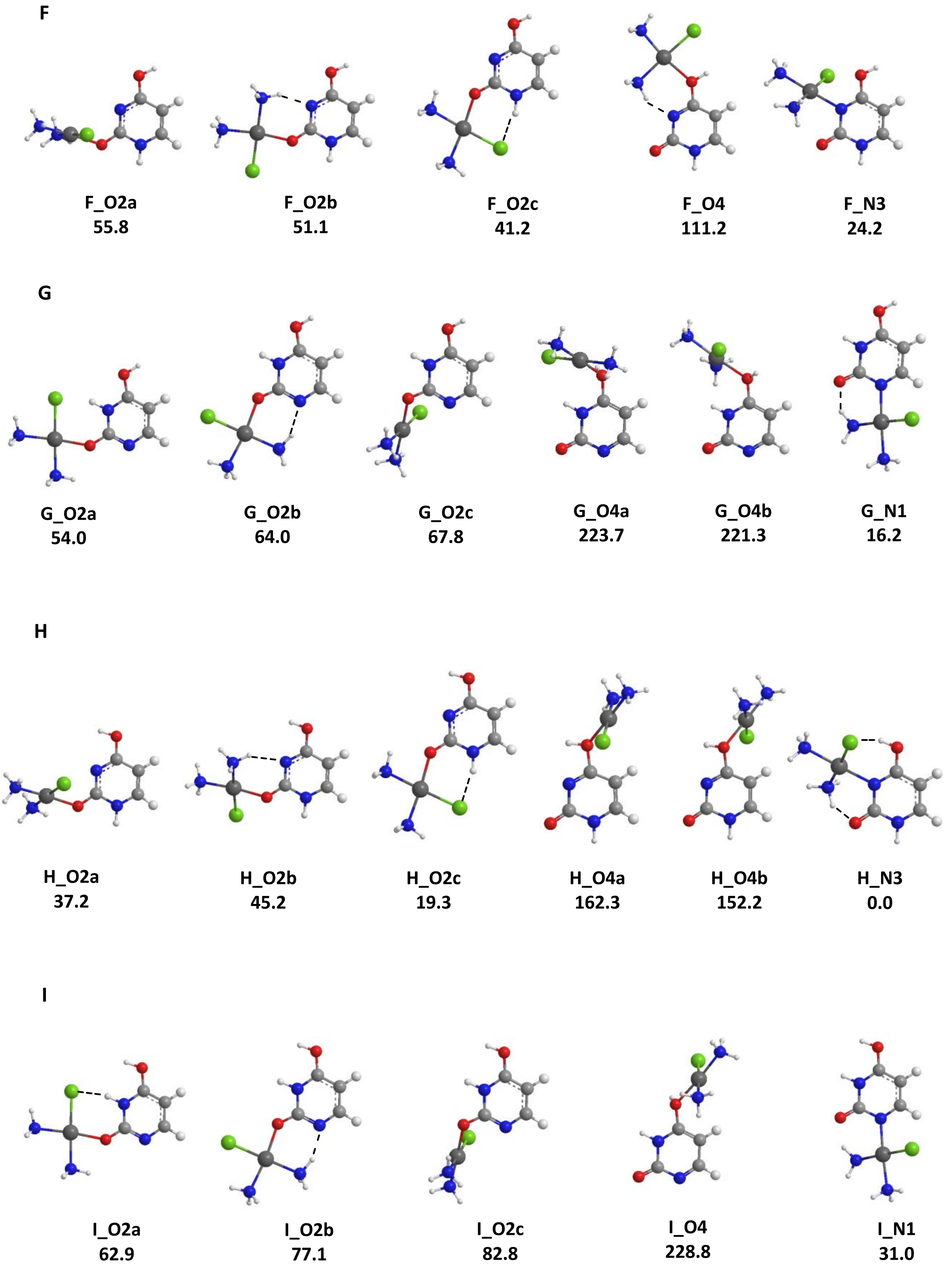

Figure S11b. Optimized geometries and relative free energy values (at the B3LYP/LACV3P/6-311G** level) at 298 K (kJ mol^{-1}) of *cis*-$[PtCl(NH_3)_2(U)]^+$ conformer and isomer families F, G, H, I. Noncovalent interactions are marked by dashed lines. Distances are given in Å.

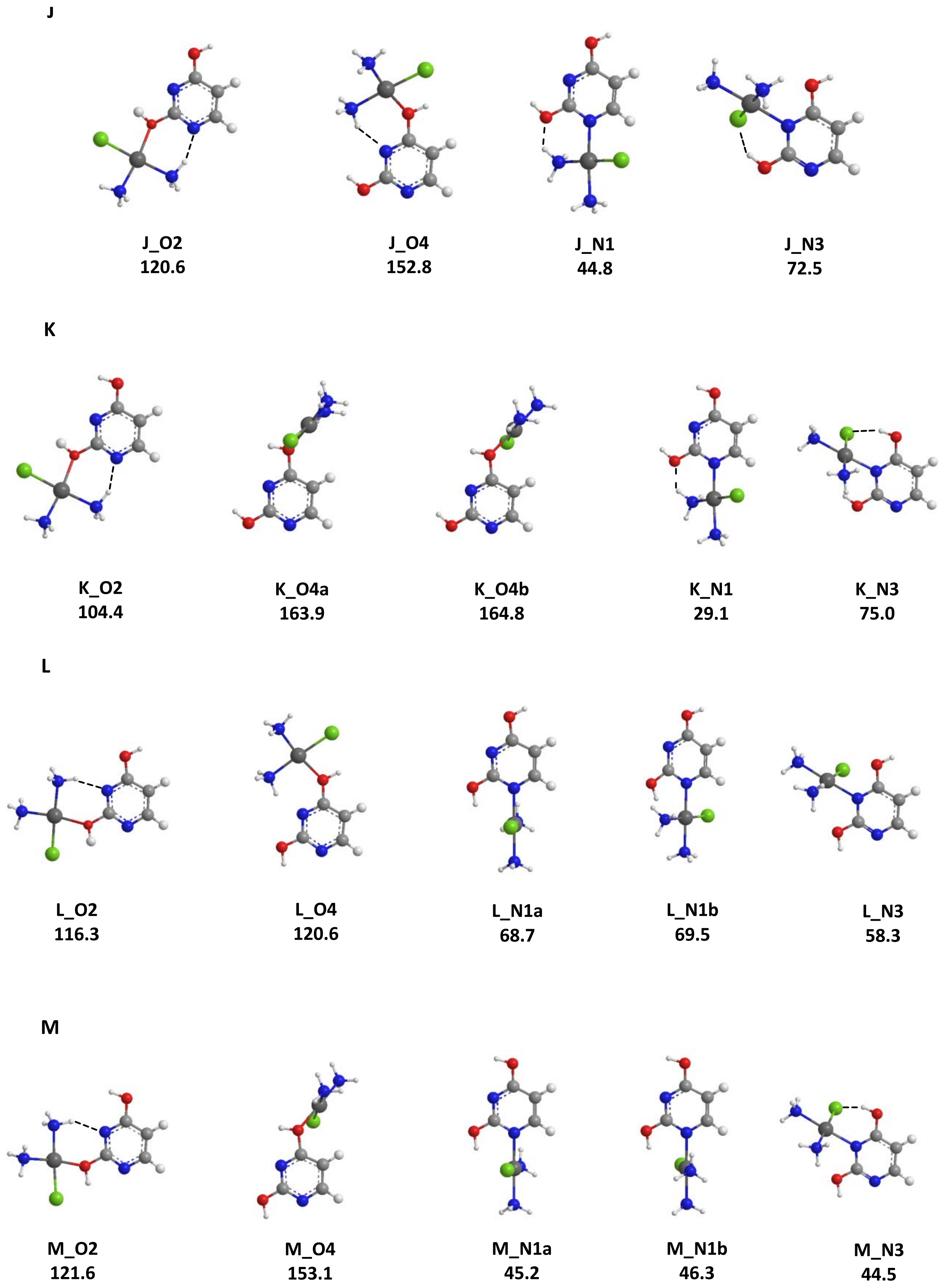

Figure S11c. Optimized geometries and relative free energy values (at the B3LYP/LACV3P/6-311G** level) at 298 K (kJ mol^{-1}) of *cis*-[PtCl(NH$_3$)$_2$(U)]$^+$ conformer and isomer families J, K, L, M. Noncovalent interactions are marked by dashed lines. Distances are given in Å.

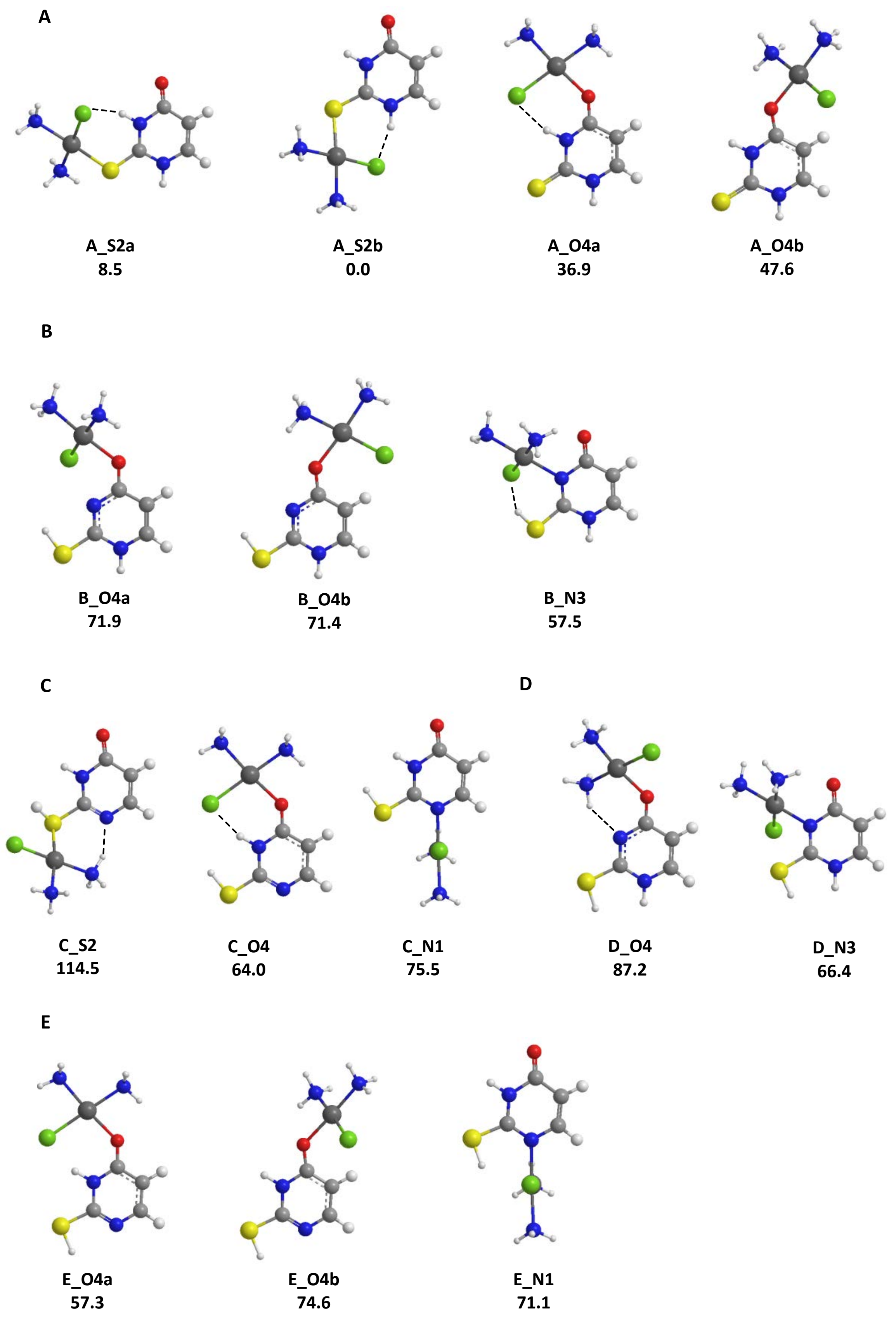

Figure S12a. Optimized geometries and relative free energy values (at the B3LYP/LACV3P/6-311G** level) at 298 K (kJ mol^{-1}) of *cis*-$[PtCl(NH_3)_2(2SU)]^+$ conformer and isomer families A, B, C, D, E. Noncovalent interactions are marked by dashed lines. Distances are given in Å.

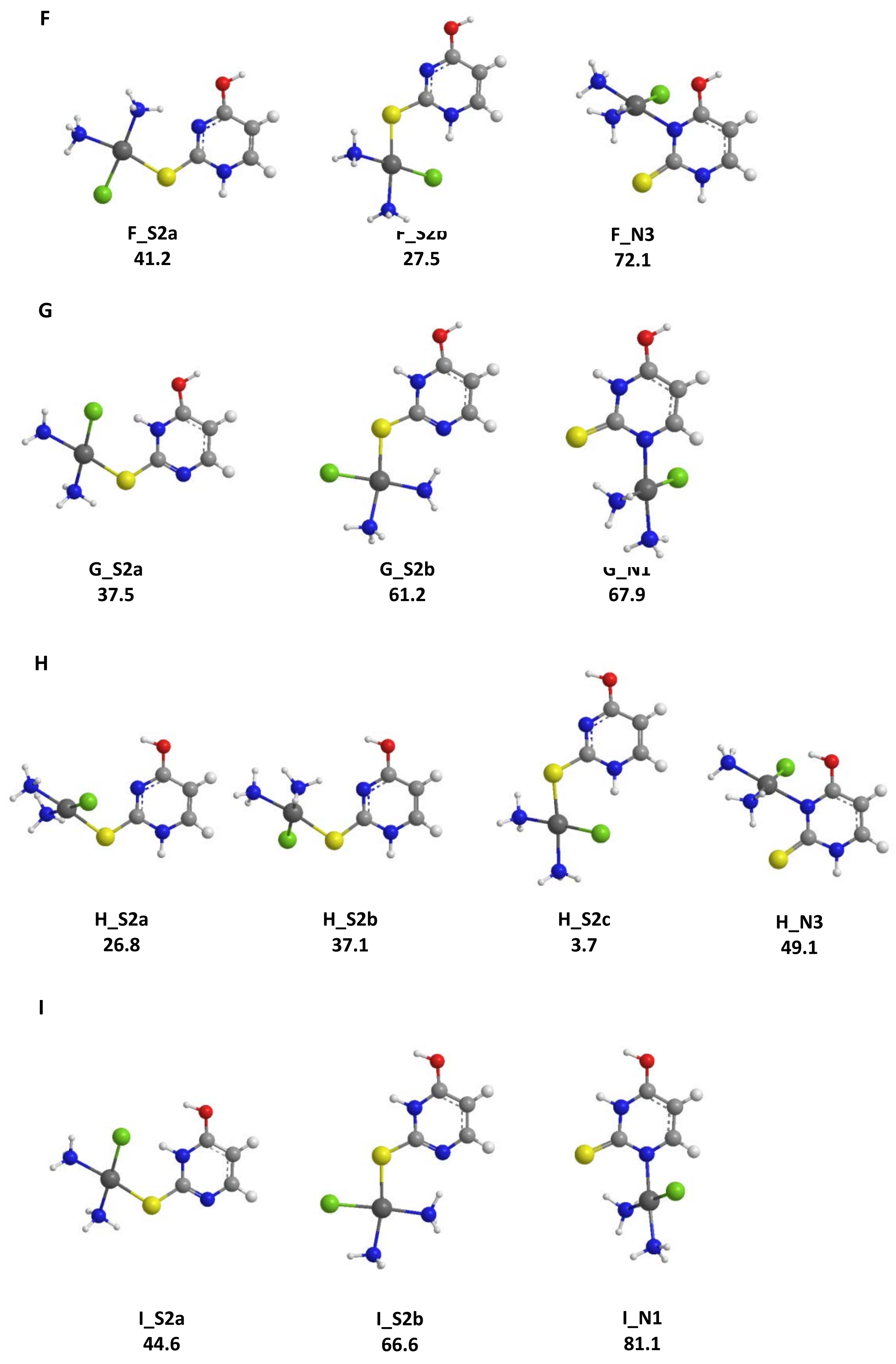

Figure S12b. Optimized geometries and relative free energy values (at the B3LYP/LACV3P/6-311G** level) at 298 K (kJ mol^{-1}) of *cis*-[PtCl(NH$_3$)$_2$(2SU)]$^+$ conformer and isomer families F, G, H, I. Noncovalent interactions are marked by dashed lines. Distances are given in Å.

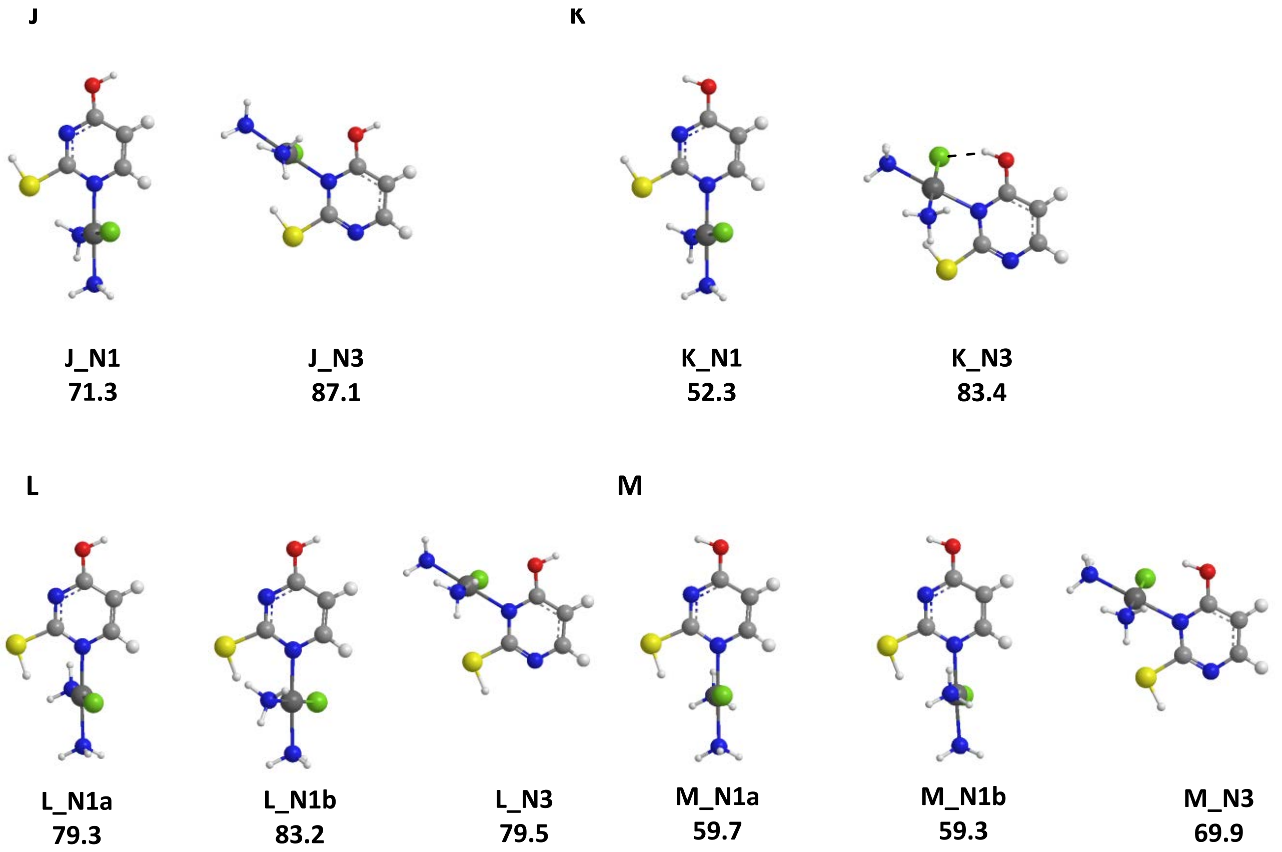

Figure S12c. Optimized geometries and relative free energy values (at the B3LYP/LACV3P/6-311G** level) at 298 K (kJ mol^{-1}) of *cis*-[PtCl$(NH_3)_2$(2SU)]$^+$ conformer and isomer families J, K, L, M. Noncovalent interactions are marked by dashed lines. Distances are given in Å.

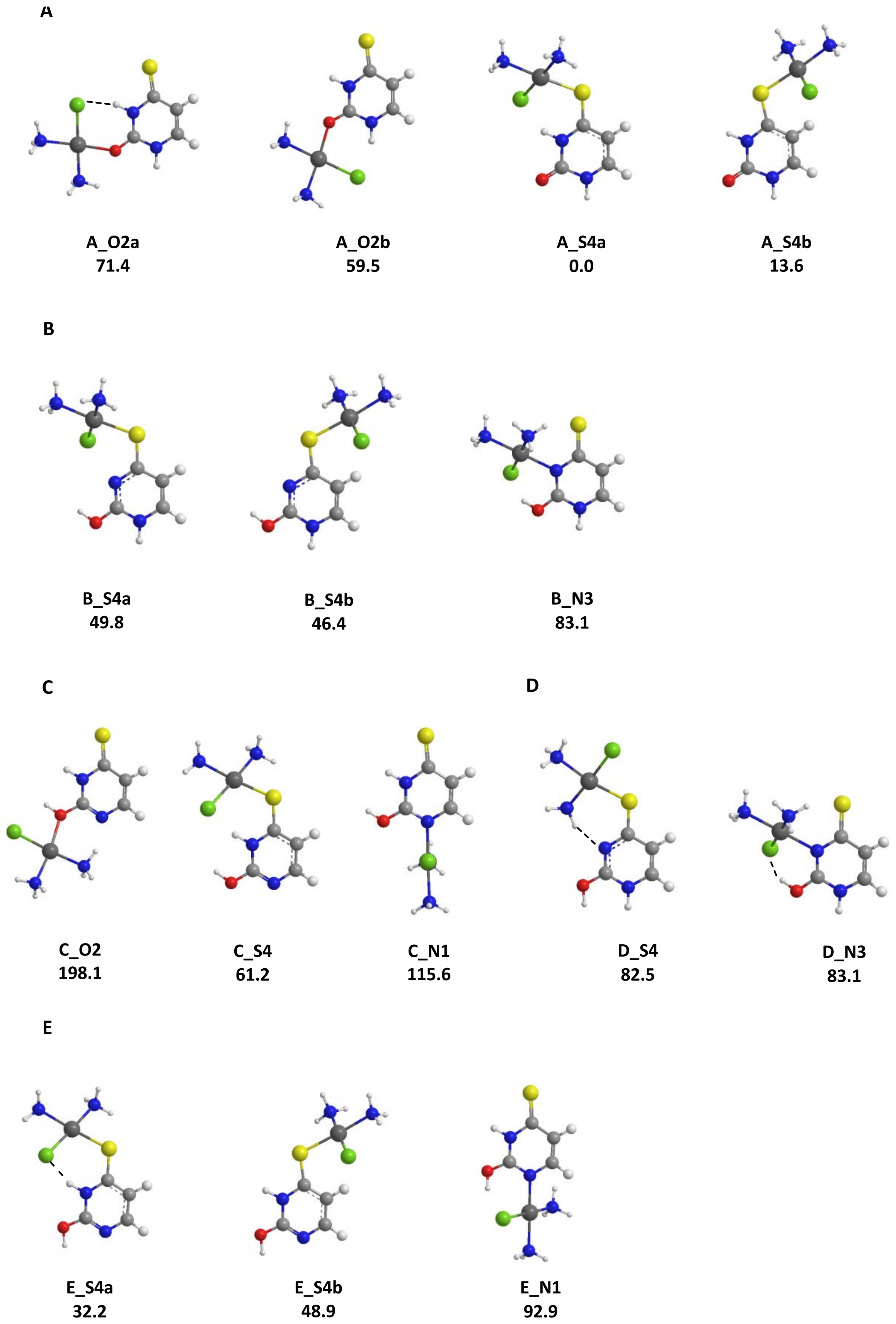

Figure S13a. Optimized geometries and relative free energy values (at the B3LYP/LACV3P/6-311G** level) at 298 K (kJ mol^{-1}) of *cis*-$[PtCl(NH_3)_2(4SU)]^+$ conformer and isomer families A, B, C, D, E. Noncovalent interactions are marked by dashed lines. Distances are given in Å.

F

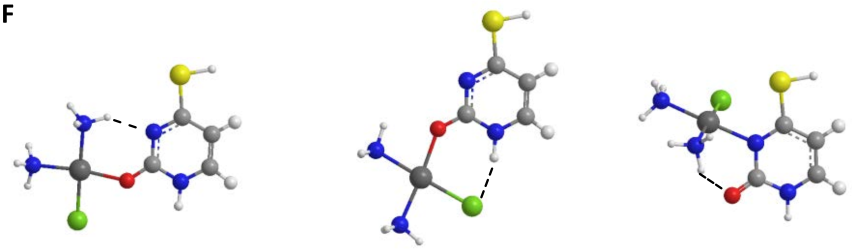

F_O2a **79.2** | **F_O2b** **64.1** | **F_N3** **54.1**

G

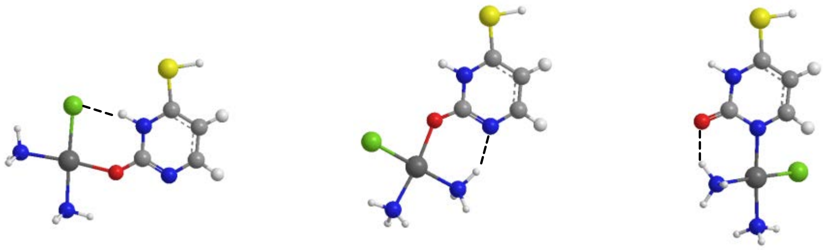

G_O2a **80.3** | **G_O2b** **91.9** | **G_N1** **44.4**

H

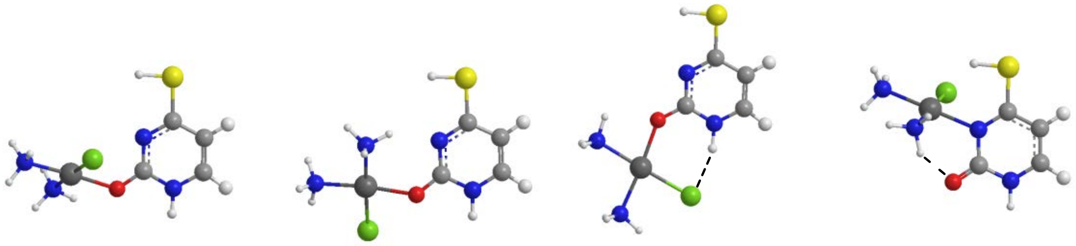

H_O2a **76.5** | **H_O2b** **80.9** | **H_O2c** **58.1** | **H_N3** **50.2**

I

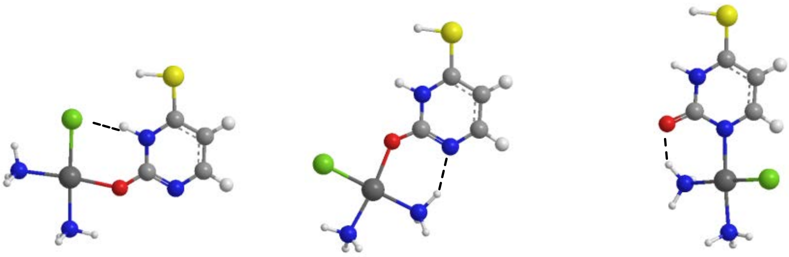

I_O2a **81.0** | **I_O2b** **94.7** | **I_N1** **47.5**

Figure S13b. Optimized geometries and relative free energy values (at the B3LYP/LACV3P/6-311G** level) at 298 K (kJ mol^{-1}) of *cis*-$[PtCl(NH_3)_2(4SU)]^+$ conformer and isomer families F, G. H, I. Noncovalent interactions are marked by dashed lines. Distances are given in Å.

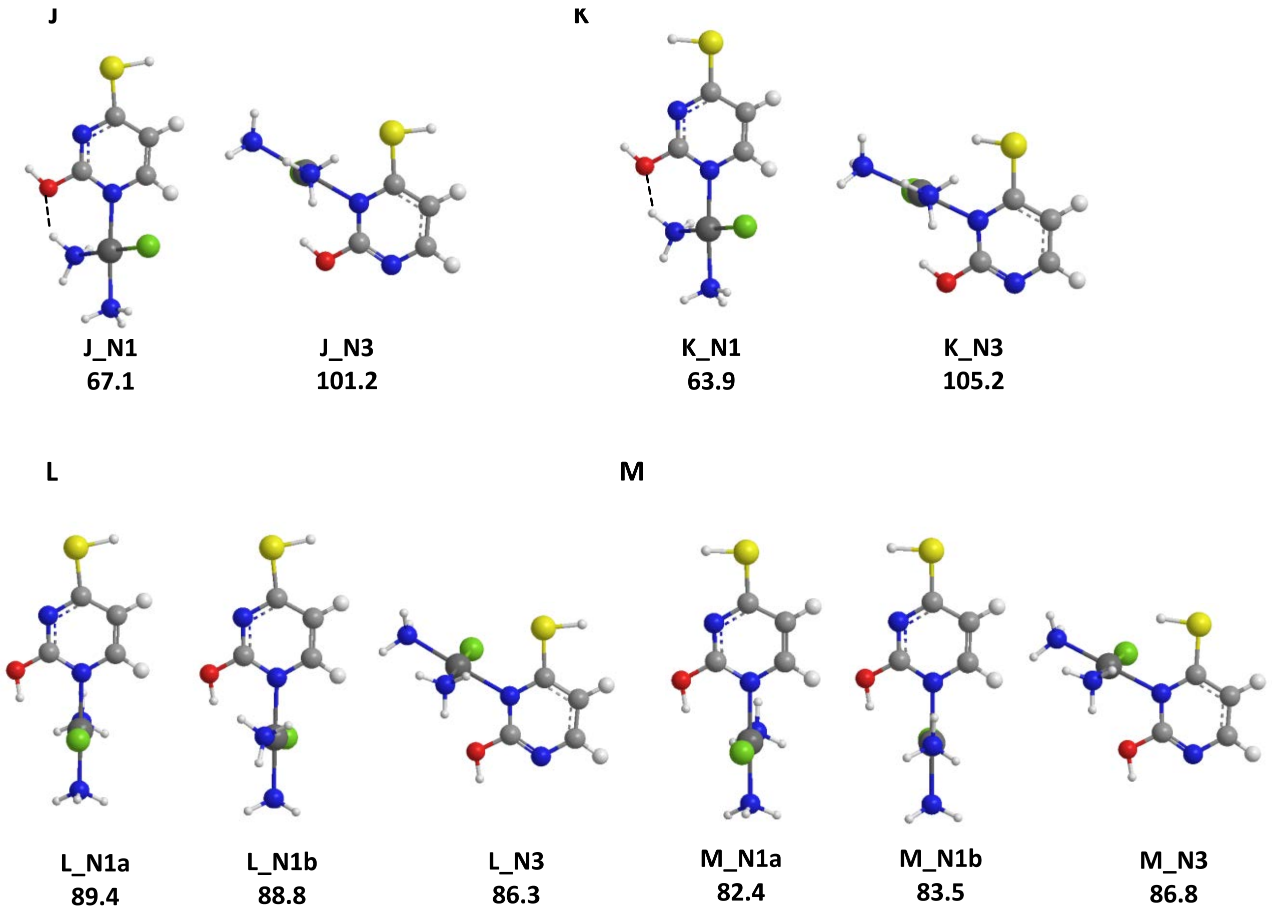

Figure S13c. Optimized geometries and relative free energy values (at the B3LYP/LACV3P/6-311G** level) at 298 K (kJ mol^{-1}) of *cis*-[PtCl(NH_3)$_2$(4SU)]$^+$ conformer and isomer families J, K, L, M. Noncovalent interactions are marked by dashed lines. Distances are given in Å.

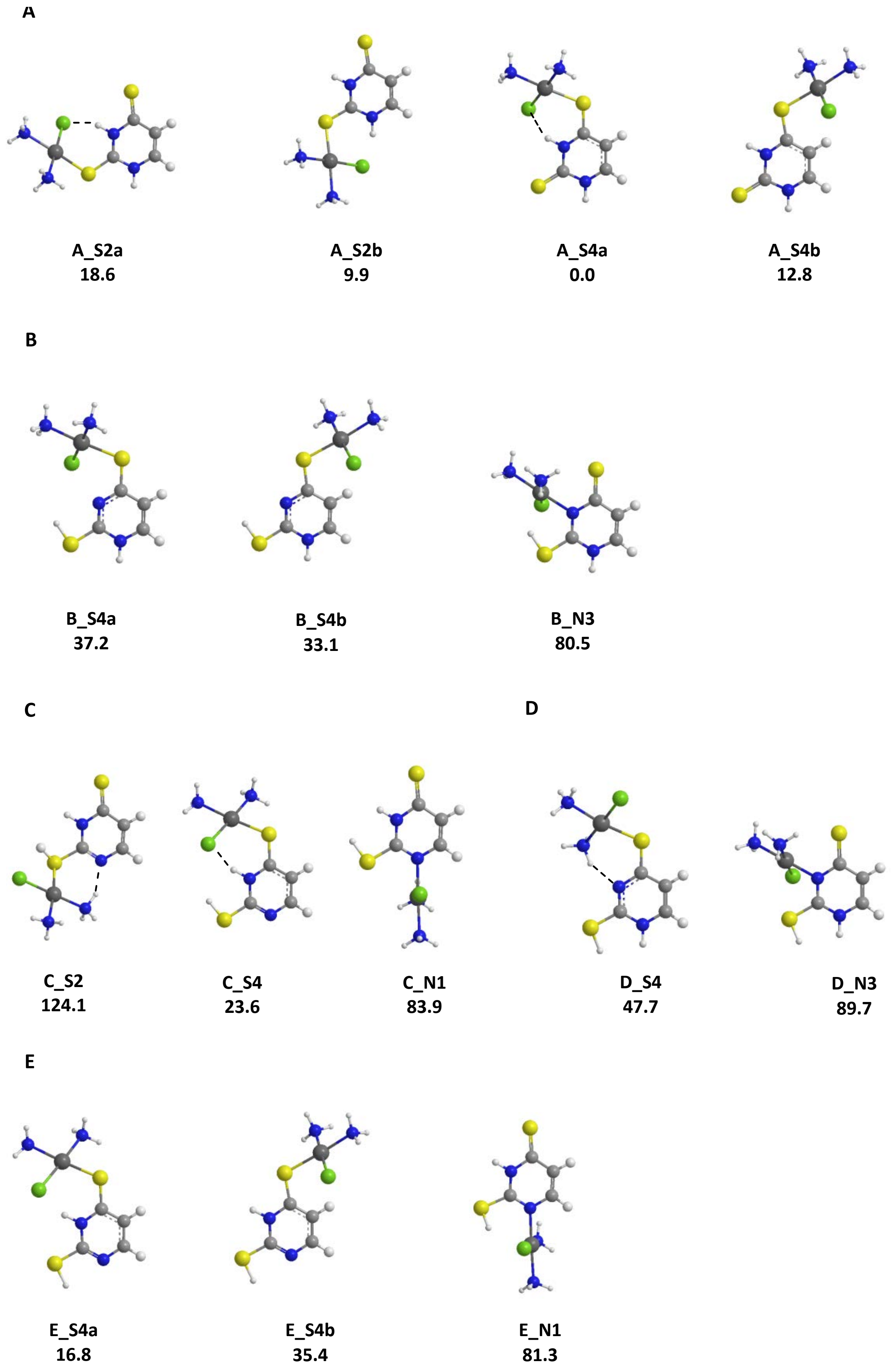

Figure S14a. Optimized geometries and relative free energy values (at the B3LYP/LACV3P/6-311G** level) at 298 K (kJ mol^{-1}) of *cis*-[PtCl$(NH_3)_2$(24dSU)]$^+$ conformer and isomer families A, B, C, D, E. Noncovalent interactions are marked by dashed lines. Distances are given in Å.

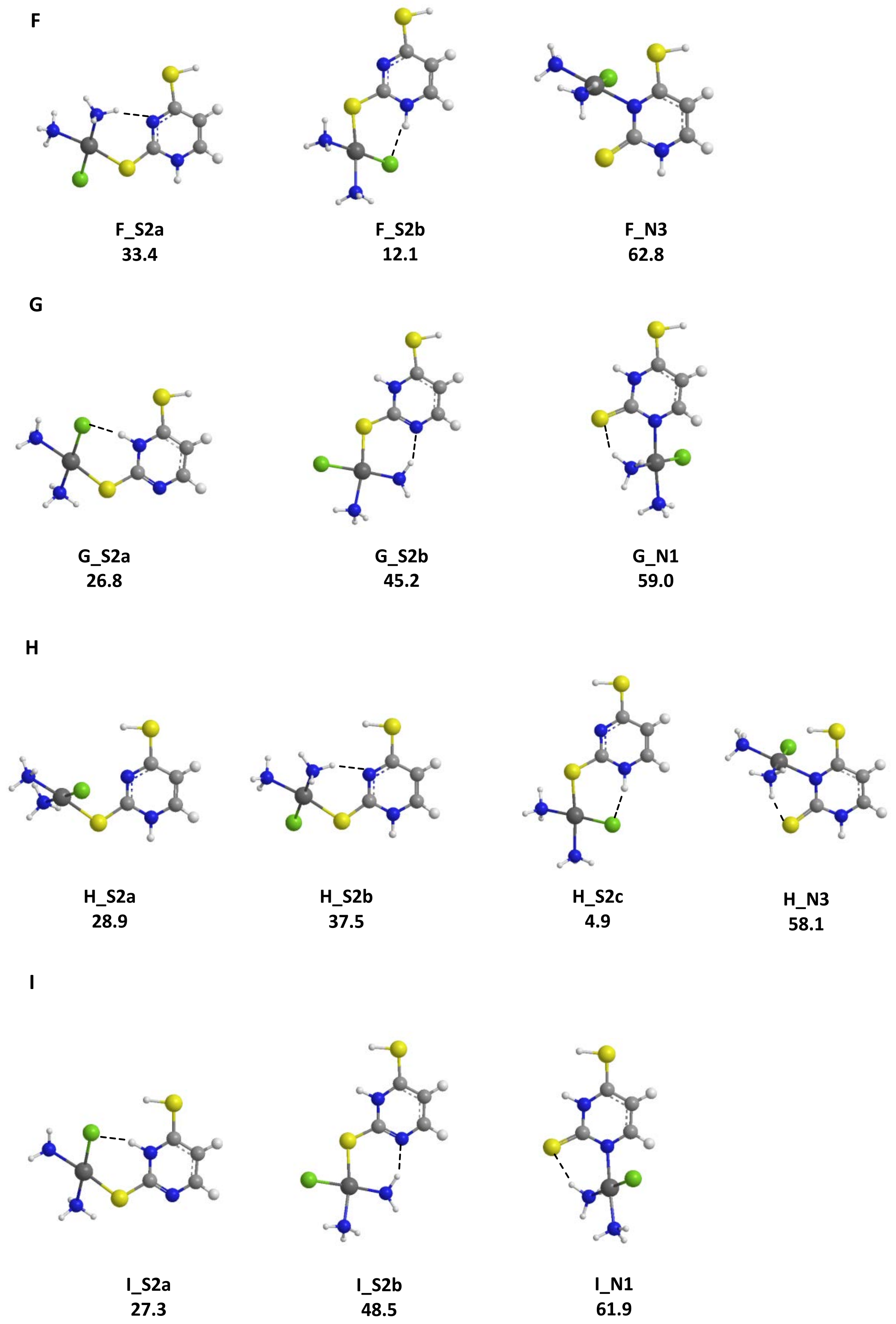

Figure S14b. Optimized geometries and relative free energy values (at the B3LYP/LACV3P/6-311G** level) at 298 K (kJ mol^{-1}) of *cis*-$[PtCl(NH_3)_2(24dSU)]^+$ conformer and isomer families F, G, H, I. Noncovalent interactions are marked by dashed lines. Distances are given in Å.

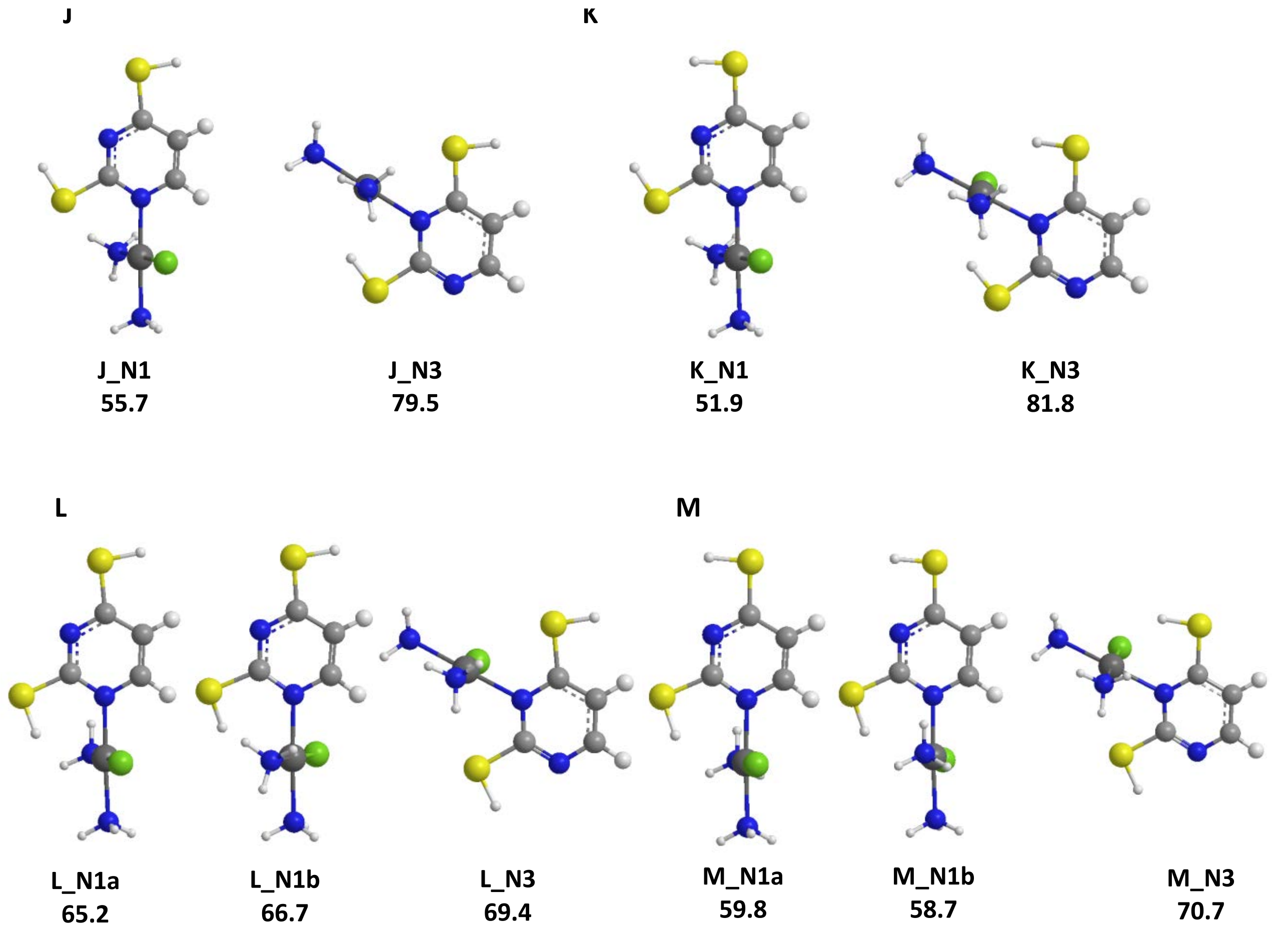

Figure S14c. Optimized geometries and relative free energy values (at the B3LYP/LACV3P/6-311G** level) at 298 K (kJ mol^{-1}) of *cis*-[PtCl$(NH_3)_2$(24dSU)]$^+$ conformer and isomer families J, K, L, M. Noncovalent interactions are marked by dashed lines. Distances are given in Å.

Table S1. Thermodynamic data for conformers and isomers of *cis*-[PtCl(NH_3)$_2$(U)]$^+$ calculated at the B3LYP/LACV3P/6-311G** level of theory.

Complex	$\Delta H°_{298}$ (kJ/mol)	$\Delta G°_{298}$ (kJ/mol)
**A_O2a**	33.0	23.7
**A_O2b**	23.4	13.8
**A_O4a**	8.5	0.0
**A_O4b**	20.2	11.9
**B_O4a**	55.6	47.6
**B_O4b**	57.3	47.8
**B_N3**	15.9	15.7
**C_O2**	160.7	151.9
**C_O4a**	73.8	66.8
**C_O4b**	99.1	90.0
**C_N1**	79.2	71.2
**D_O2**	174.7	165.9
**D_O4a**	93.8	83.2
**D_O4b**	92.6	85.0
**D_O4c**	92.9	83.6
**E_O2**	184.1	172.8
**E_O4a**	42.4	35.8
**E_O4b**	59.2	51.9
**E_N1**	53.4	48.3
**F_O2a**	66.5	55.8
**F_O2b**	58.5	51.1
**F_O2c**	48.5	41.2
**F_O4**	118.7	111.2
**F_N3**	26.9	24.2
**G_O2a**	61.1	54.0
**G_O2b**	72.2	64.0
**G_O2c**	78.5	67.8
**G_O4a**	237.8	223.7
**G_O4b**	239.4	221.3
**G_N1**	18.5	16.2
**H_O2a**	45.7	37.2
**H_O2b**	52.9	45.2
**H_O2c**	27.3	19.3
**H_O4a**	160.1	162.3
**H_O4b**	162.3	152.2
**H_N3**	0.0	0.0
**I_O2a**	70.3	62.9

**I_O2b**	86.0	77.1
**I_O2c**	95.0	82.8
**I_O4**	242.9	228.8
**I_N1**	33.9	31.0
**J_O2**	127.3	120.6
**J_O4**	160.7	152.8
**J_N1**	48.7	44.8
**J_N3**	77.7	72.5
**K_O2**	110.9	104.4
**K_O4a**	175.2	163.9
**K_O4b**	175.1	164.8
**K_N1**	33.0	29.1
**K_N3**	77.9	75.0
**L_O2**	124.0	116.3
**L_O4**	130.5	120.6
**L_N1a**	76.5	68.7
**L_N1b**	77.4	69.5
**L_N3**	62.6	58.3
**M_O2**	129.0	121.6
**M_O4**	164.9	153.1
**M_N1a**	52.9	45.2
**M_N1b**	53.0	46.3
**M_N3**	46.7	44.5

Table S2. Thermodynamic data for conformers and isomers of *cis*-$[PtCl(NH_3)_2(L)]^+$ (L=2SU, 4SU and 24dSU) calculated at the B3LYP/LACV3P/6-311G** level of theory.

Complex	ΔH°$_{298}$ (kJ/mol)	ΔG°$_{298}$ (kJ/mol)
	2SU	
**A_S2a**	7.2	8.5
**A_S2b**	0.0	0.0
**A_O4a**	34.3	36.9
**A_O4b**	44.04	47.6
**B_O4a**	70.5	71.9
**B_O4b**	71.9	71.4
**B_N3**	48.5	57.5
**C_S2**	109.9	114.5
**C_O4**	61.2	64.0
**C_N1**	74.2	75.5
**D_O4**	85.3	87.2
**D_N3**	59.9	66.4
**E_O4a**	54.1	57.3
**E_O4b**	72.5	74.6
**E_N1**	71.9	71.1
**F_S2a**	38.1	41.2
**F_S2b**	23.9	27.5
**F_N3**	63.7	72.1
**G_S2a**	35.9	37.5
**G_S2b**	49.2	61.2
**G_N1**	60.2	67.9
**H_S2a**	26.0	26.8
**H_S2b**	33.8	37.8
**H_S2c**	1.1	3.7
**H_N3**	40.3	49.1
**I_S2a**	42.8	44.6
**I_S2b**	66.4	66.6
**I_N1**	73.9	81.1
**J_N1**	67.0	71.3
**J_N3**	85.4	87.1
**K_N1**	48.6	52.3
**K_N3**	76.7	83.4
**L_N1a**	79.5	79.3
**L_N1b**	78.8	83.2
**L_N3**	75.6	79.5
**M_N1a**	57.8	59.7
**M_N1b**	57.7	59.3
**M_N3**	64.5	69.9

4SU		
**A_O2a**	71.4	71.4
**A_O2b**	60.0	59.5
**A_S4a**	0.0	0.0
**A_S4b**	14.9	13.6
**B_S4a**	50.5	49.8
**B_S4b**	47.8	46.4
**B_N3**	75.5	83.1
**C_O2**	198.0	198.1
**C_S4**	61.3	61.2
**C_N1**	116.7	115.6
**D_S4**	81.1	82.5
**D_N3**	75.6	83.1
**E_S4a**	31.4	32.2
**E_S4b**	49.8	48.9
**E_N1**	88.7	92.9
**F_O2a**	79.3	79.2
**F_O2b**	62.9	64.1
**F_N3**	48.4	54.1
**G_O2a**	80.1	80.3
**G_O2b**	92.6	91.9
**G_N1**	39.2	44.4
**H_O2a**	77.3	76.5
**H_O2b**	81.8	80.9
**H_O2c**	58.4	58.1
**H_N3**	43.7	50.2
**I_O2a**	81.2	81.0
**I_O2b**	95.8	94.7
**I_N1**	43.2	47.5
**J_N1**	62.9	67.1
**J_N3**	100.3	101.2
**K_N1**	60.0	63.9
**K_N3**	104.9	105.2
**L_N1a**	89.3	89.4
**L_N1b**	90.4	88.9
**L_N3**	82.7	86.3
**M_N1a**	82.7	82.4
**M_N1b**	82.9	83.5
**M_N3**	82.4	86.8
24dSU		
**A_S2a**	20.2	18.6
**A_S2b**	11.8	9.9

**A_S4a**	0.0	0.0
**A_S4b**	14..0	12.8
**B_S4a**	39.9	37.2
**B_S4b**	36.4	33.1
**B_N3**	74.8	80.5
**C_S2**	121.5	124.1
**C_S4**	24.4	23.6
**C_N1**	85.1	83.9
**D_S4**	48.8	47.7
**D_N3**	85.8	89.7
**E_S4a**	17.6	16.8
**E_S4b**	38.2	35.4
**E_N1**	82.7	81.3
**F_S2a**	33.8	33.4
**F_S2b**	11.9	12.1
**F_N3**	57.4	62.8
**G_S2a**	29.2	26.8
**G_S2b**	47.6	45.2
**G_N1**	55.4	59.0
**H_S2a**	32.1	28.9
**H_S2b**	37.1	37.5
**H_S2c**	6.1	4.9
**H_N3**	51.9	58.1
**I_S2a**	29.3	27.3
**I_S2b**	50.9	48.5
**I_N1**	58.5	61.9
**J_N1**	55.0	55.7
**J_N3**	79.6	79.5
**K_N1**	51.2	51.9
**K_N3**	82.6	81.8
**L_N1a**	66.8	65.2
**L_N1b**	66.1	66.7
**L_N3**	68.9	69.4
**M_N1a**	61.2	59.8
**M_N1b**	61.0	58.7
**M_N3**	61.0	70.7

Table S3. Experimental and computed IR vibrational bands for cis-[PtCl(NH_3)$_2$(U)]$^+$.

U_A_O4a

Wavenumbers		DFT-computed	Vibrational mode[b]
Exp.	Calc.[a]		
1209	1205	92	δ N1H + δ CH
1290	1288	131	δ NH_3' umbrella
	1324	99	δ NH_3 umbrella
	1386	118	δ N3H
1426	1424	73	δ N1H
1480	1513	118	ν C4C5 + ν N1C6 + δ N1H
1580	1585	226	ν C4O4
1615	1630	986	ν C5C6
	1634	59	δ NH of NH_3 and NH_3'
1800	1817	600	ν C2O2
	3183	580	ν N3H
	3346	58	ν NH_2 asym of NH_3
	3373	74	ν NH_2 asym of NH_3'
	3384	46	ν NH_2 asym of NH_3
	3394	55	ν NH_2 asym of NH_3'
3450	3448	185	ν N1H

a) Scaled by a factor of 0.974 in the fingerprint region and 0.957 in the X-H stretch region
b) NH_3' is the NH_3 trans to Cl

U_A_O4b

Wavenumbers		DFT-computed	Vibrational mode[b]
Exp.	Calc.[a]		
1209	1210	109	δ N1H + δ CH
1290	1289	119	δ NH_3' umbrella
	1319	104	δ NH_3 umbrella
	1367	68	δ CH
1426	1417	71	δ N1H + ν C4C5
1480	1504	210	δ N1H + ν N3C4
1580	1571	361	ν C4O4 + δ N3H
	1615	179	δ NH_2 sciss. of NH_3'
1615	1624	720	ν C5C6
	1635	53	δ NH of NH_3 and NH_3'
1800	1819	764	ν C2O2
	3345	61	ν NH_2 asym of NH_3

	3379	59	ν NH_2 asym of NH_3'
	3386	40	ν NH_2 asym of NH_3
	3392	55	ν NH_2 asym of NH_3'
	3580	100	ν N3H
3450	3446	188	ν N1H

a) Scaled by a factor of 0.974 in the fingerprint region and 0.957 in the X-H stretch region
b) NH_3' is the NH_3 trans to Cl

U_A_O2b

Wavenumbers		DFT-computed	Vibrational mode[b]
Exp.	Calc.[a]		
	1167	**76**	δ C5H + ν N3C4
1209	1212	47	δ CH
1290	1297	116	δ NH_3' umbrella
	1328	89	δ NH_3 umbrella
	1348	48	δ N3H
1426	-		
1480	1466	148	ν N1C2
1580	-		
1615	-		
	1635	59	δ NH of NH_3 and NH_3'
	1639	910	ν C2O2 + ν C5C6
	1656	547	ν C5C6 + δ N1H
1800	1786	500	ν C4O4
	3209	764	ν N1H
	3346	67	ν NH_2 asym of NH_3
	3373	71	ν NH_2 asym of NH_3'
	3381	46	ν NH_2 asym of NH_3
	3391	59	ν NH_2 asym of NH_3'
3450	3435	78	ν N3H

a) Scaled by a factor of 0.974 in the fingerprint region and 0.957 in the X-H stretch region
b) NH_3' is the NH_3 trans to Cl

Table S4: Experimental and computed IR vibrational bands for the *cis*-$[PtCl(NH_3)_2(2SU)]^+$ complex

2SU_A_S2b

Wavenumbers		DFT-computed	Vibrational mode[b]
Exp.	Calc.[a]		
1145-1240	1170	170	δ C5H + ν N3C4 + ν C2S2
	1214	85	δ C6H
	1286	124	δ NH_3 umbrella
1297	1305	117	δ NH_3' umbrella
1488	1463	80	ν N1C2
1560	1563	596	δ N1H
	1603	40	δ NH_2 sciss. of NH_3
1619	1637	53	δ NH of both NH_3
	1643	40	ν C5C6
1770	1790	608	ν C4O4
	3154	689	ν N1H
	3362	49	ν NH_2 asym of NH_3
	3367	68	ν NH_2 asym of NH_3'
	3387	37	ν NH_2 asym of NH_3'
	3395	54	ν NH_2 asym of NH_3
3405	3409	86	ν N3H
3569	-		

a) Scaled by a factor of 0.974 in the fingerprint region and 0.957 in the X-H stretch region
b) NH_3' is the NH_3 trans to Cl

2SU_A_S2a

Wavenumbers		DFT-computed	Vibrational mode[b]
Exp.	Calc.[a]		
1145-1240	1178	195	δ C5H + ν N3C4 + ν C2S2
	1204	87	δ C6H
1297	1285	126	δ NH_3 umbrella
	1298	112	δ NH_3' umbrella
1488	1465	175	ν N1C2
1560	1554	540	δ N1H + δ N3H
1619	1603	37	δ NH_2 sciss. of NH_3
	1618	28	δ NH_2 sciss. of NH_3'
	1637	53	δ NH of NH_3
	1641	47	ν C5C6
1770	1790	437	ν C4O4

	3242	314	ν N3H
	3286	34	ν NH_3 sym
	3359	54	ν NH_2 asym of NH_3
	3372	69	ν NH_2 asym of NH_3’
	3388	36	ν NH_2 asym of NH_3’
3405	3396	53	ν NH_2 asym of NH_3
	3452	127	ν N1H
3569	-		

a) Scaled by a factor of 0.974 in the fingerprint region and 0.957 in the X-H stretch region
b) NH_3’ is the NH_3 *trans* to Cl

2SU_H_S2c

Wavenumbers		DFT-computed	Vibrational mode[b]
Exp.	Calc.[a]		
1145-1240	1136	398	δ O4H
	1253	72	ν N1C2 + δ O4H
1297	1279	114	δ NH_3 umbrella
	1297	143	δ NH_3’ umbrella
	1329	83	ν N3C2 + δ CH
	1460	329	ν N3C4 + ν C4O4
1488	1515	240	δ N1H + ν C4C5
1560	1584	634	δ N1H+ ν N3C4
1619	1617	33	δ NH_2 sciss. of NH_3 and NH_3'
	1626	215	ν C5C6
	1628	42	δ NH_2 twist. of NH_3 and NH_3'
	1636	52	δ NH of NH_3 and NH_3'
1770	-		
	3106	587	ν N1H
	3364	55	ν NH_2 asym of NH_3’
	3366	62	ν NH_2 asym of NH_3
	3390	36	ν NH_2 asym of NH_3’
3405	3397	49	ν NH_2 asym of NH_3
3569	3578	155	ν OH

a) Scaled by a factor of 0.974 in the fingerprint region and 0.957 in the X-H stretch region
b) NH_3’ is the NH_3 *trans* to Cl

Table S5: Experimental and computed IR vibrational bands for the *cis*-[PtCl(NH_3)$_2$(4SU)]$^+$ complex.

4SU_A_S4a

Wavenumbers		DFT-computed	Vibrational mode[b]
Exp.	Calc.[a]		
1098	1084	131	ν C4S4 + ν N1C2
1193	1170	144	ν C2N3
1279	1283	129	δ NH_3 umbrella
	1298	120	δ NH_3' umbrella
	1413	84	δ N1H
1483	1500	56	δ N3H
	1512	84	δ N1H + δ N3H + ν C2O2
1609	1603	463	ν C5C6
	1604	162	δ NH_2 sciss. of NH_3
	1619	57	δ NH_2 sciss. of NH_3'
	1638	48	δ NH of NH_3 and of NH_3'
1808	1817	650	ν C2O2
	3222	330	ν N3H
	3361	54	ν NH_2 asym of NH_3
	3372	63	ν NH_2 asym of NH_3'
	3390	36	ν NH_2 asym of NH_3'
	3397	49	ν NH_2 asym of NH_3
3452	3441	230	ν N1H

a) Scaled by a factor of 0.974 in the fingerprint region and 0.957 in the X-H stretch region
b) NH_3' is the NH_3 trans to Cl

4SU_A_S4b

Wavenumbers		DFT-computed	Vibrational mode[b]
Exp.	Calc.[a]		
1098	1093	113	ν C4S4 + δ C5H
	1094	102	ring breathing
1193	-		
1279	1279	130	δ NH_3 umbrella
	1296	119	δ NH_3' umbrella
	1408	78	δ N1H + ν N3C4
1483	1477	167	δ N3H
	1509	71	δ N1H + ν C6N1
1609	1597	593	ν C5C6
	1602	36	δ NH_2 sciss. of NH_3

	1619	46	δ NH_2 sciss. of NH_3’
	1649	49	δ NH of NH_3 and of NH_3’
1808	1820	914	ν C2O2
	3360	57	ν NH_2 asym of NH_3
	3376	69	ν NH_2 asym of NH_3’
	3391	35	ν NH_2 asym of NH_3’
	3399	48	ν NH_2 asym of NH_3
	3406	93	ν N3H
3452	3440	235	ν N1H

a) Scaled by a factor of 0.974 in the fingerprint region and 0.957 in the X-H stretch region
b) NH_3’ is the NH_3 *trans* to Cl

Table S6: Experimental and computed IR vibrational bands for the *cis*-[PtCl(NH_3)$_2$(24dSU)]$^+$ complex.

24dSU_A_S4a

Wavenumbers		DFT-computed	Vibrational mode[b]
Exp.	Calc.[a]		
1094	1078	149	ν C4S4
	1095	154	ring breathing
1123	-		
1163	-		
1194	1184	197	ν N2C3
	1208	60	δ CH + ring breathing
1280	1283	134	δ NH_3 umbrella
	1297	123	δ NH_3’ umbrella
1445	-		
	1496	73	δ N3H
1554	1556	618	δ N1H + δ N3H + ν C2S2
1586	1591	641	ν C5C6
	1619	42	δ NH_2 sciss. of NH_3’
	1637	51	δ NH_2 sciss. of NH_3
	3224	304	ν N3H
3285	3271	27	ν NH_3 sym.
	3290	16	ν NH_3’ sym.
3380	3361	55	ν NH_2 asym of NH_3
	3373	64	ν NH_2 asym of NH_3’
	3390	35	ν NH_2 asym of NH_3’
	3397	50	ν NH_2 asym of NH_3
3431	3434	189	ν N1H
3452	-		

a) Scaled by a factor of 0.974 in the fingerprint region and 0.957 in the X-H stretch region
b) NH_3’ is the NH_3 *trans* to Cl

24dSU_A_S4b

Wavenumbers		DFT-computed	Vibrational mode[b]
Exp.	Calc.[a]		
1094	1076	173	δ N3H + ν C4S4
	1096	316	ring breathing
1123	-		
1163	-		
1194	1185	161	ν N2C3

	1208	47	ring breathing
1280	1279	128	δ NH_3 umbrella
	1292	124	δ NH_3' umbrella
1445	-		
	1491	130	δ N3H
1554	1550	961	δ N1H + δ N3H + ν C2S2
1586	1585	624	ν C5C6
	1602	36	δ NH_2 sciss. of NH_3
	1619	41	δ NH_2 sciss. of NH_3'
	1638	46	δ NH of NH_3 and of NH_3'
3285	3266	25	ν NH_3 sym
	3293	15	ν NH_2 asym of NH_3
3380	3376	63	ν NH_2 asym of NH_3'
	3391	35	ν NH_2 asym of NH_3'
	3398	48	ν NH_2 asym of NH_3
	3399	70	ν N3H
3431	3434	189	ν N1H
3452	-		

a) Scaled by a factor of 0.974 in the fingerprint region and 0.957 in the X-H stretch region
b) NH_3' is the NH_3 *trans* to Cl

Complete reference for Gaussian 09

Gaussian 09, Revision D.01, M. J. Frisch, G. W. Trucks, H. B. Schlegel, G. E. Scuseria, M. A. Robb, J. R. Cheeseman, G. Scalmani, V. Barone, B. Mennucci, G. A. Petersson, H. Nakatsuji, M. Caricato, X. Li, H. P. Hratchian, A. F. Izmaylov, J. Bloino, G. Zheng, J. L. Sonnenberg, M. Hada, M. Ehara, K. Toyota, R. Fukuda, J. Hasegawa, M. Ishida, T. Nakajima, Y. Honda, O. Kitao, H. Nakai, T. Vreven, J. A. Montgomery, Jr., J. E. Peralta, F. Ogliaro, M. Bearpark, J. J. Heyd, E. Brothers, K. N. Kudin, V. N. Staroverov, R. Kobayashi, J. Normand, K. Raghavachari, A. Rendell, J. C. Burant, S. S. Iyengar, J. Tomasi, M. Cossi, N. Rega, J. M. Millam, M. Klene, J. E. Knox, J. B. Cross, V. Bakken, C. Adamo, J. Jaramillo, R. Gomperts, R. E. Stratmann, O. Yazyev, A. J. Austin, R. Cammi, C. Pomelli, J. W. Ochterski, R. L. Martin, K. Morokuma, V. G. Zakrzewski, G. A. Voth, P. Salvador, J. J. Dannenberg, S. Dapprich, A. D. Daniels, Ö. Farkas, J. B. Foresman, J. V. Ortiz, J. Cioslowski, and D. J. Fox, Gaussian, Inc., Wallingford CT, 2009.
